# The Airport Gate Assignment Problem: A Survey

**DOI:** 10.1155/2014/923859

**Published:** 2014-11-20

**Authors:** Abdelghani Bouras, Mageed A. Ghaleb, Umar S. Suryahatmaja, Ahmed M. Salem

**Affiliations:** Industrial Engineering Department, College of Engineering, King Saud University, P.O. Box 800, Riyadh 11421, Saudi Arabia

## Abstract

The airport gate assignment problem (AGAP) is one of the most important problems operations managers face daily. Many researches have been done to solve this problem and tackle its complexity. The objective of the task is assigning each flight (aircraft) to an available gate while maximizing both conveniences to passengers and the operational efficiency of airport. This objective requires a solution that provides the ability to change and update the gate assignment data on a real time basis. In this paper, we survey the state of the art of these problems and the various methods to obtain the solution. Our survey covers both theoretical and real AGAP with the description of mathematical formulations and resolution methods such as exact algorithms, heuristic algorithms, and metaheuristic algorithms. We also provide a research trend that can inspire researchers about new problems in this area.

## 1. Introduction

The complexity of airport management has increased significantly. Flight delays or accidents might happen if operations were not handled well, and domino effect might happen to influence the whole operations of airport. In airports, the tasks related to gate assignment problem (AGAP) are one of the most important daily operations many researches have been published on with the aim of solving the problem in spite of its complexity. The objective of the task is assigning each flight (aircraft) to an available gate while maximizing both conveniences to passengers and the operational efficiency of airport. Large airlines typically need to manage different gates across an airport in the most efficient way in a dynamic operational environment. This requires a solution that provides the ability to change and update the gate assignment data on a real time basis. It should also provide robust and efficient disruption management, while maintaining safety, security, and cost efficiency.

Numerous methods have been developed to solve this problem since 1974. Steuart [1] proposed simple stochastic model to find the efficiency use of the gate positions. The research interest in this field was slow in development because there were less than 15 publications within 25 years. However, after 2000, the interest to develop solutions for this problem increased, until nowadays, though with small growth. The objective of this problem varied and depended on the point of view. The first is as an airport owner, which is the government. The objectives are to maximize the utilization of the available gates and terminal [1–4], minimize the number of gate conflicts [5], minimize the number of ungated flights [3, 6–9], and minimize the flights delay [10]. Another point of view is as an airlines owner. Their goals were to increase the customer satisfaction with minimizing the passenger walking distance between gates [3, 6, 7, 11–18] and minimizing the travelling distance from runway to the gate [19].

Dorndorf et al. [20] divided the objectives into five parts, which are reducing the number of the procedures for the costly aircraft towing, minimizing the passengers total walking distance, minimizing the deviations in the schedules, minimizing the number of ungated aircrafts, and maximizing the preferences (i.e., certain aircrafts should go for particular gates). They also defined three usually used constraints, which are the fact that only one aircraft can be gated in a defined amount of time, the fulfillment of the space restriction and service requirements, and the assurance of getting a minimum time between sequent aircrafts and a minimum ground time.

The solution approaches and the solving techniques are varied with no methods, until nowadays, that provide a robust technique for such problem. This study focuses on assessing the trend of solving gate assignment problem in light of the preceding four points. Specifically, this study will address the following research questions. (1) Is this problem NP-hard? (2) What formulation can be defined for such problem? (3) How effective are the recent methods and techniques to solve the problem? (4) What recommendation can be made based on the current findings with regard to research trends?

From a mathematical view, AGAP has been formulated as integer, binary, or mixed integer, general linear or nonlinear models. Specific formulation as binary or mixed binary quadratic models has also been suggested. Other well-known related problems in combinatorial optimization such as quadratic assignment problem (QAP), clique partitioning problem (CPP), and scheduling problem have been used to formulate AGAP. However, few publications on AGAP tackled stochastic or robust optimization.

While the goal of combinatorial optimization research is to find an algorithm that guarantees an optimal solution in polynomial time with respect to the problem size, the main interest in practice is to find a nearly optimal or at least good-quality solution in a reasonable amount of time. Many approaches to solve the GAP have been proposed, varying from Brand and Bound (B&B) to highly esoteric optimization methods. The majority of these methods can be broadly classified as either “exact” algorithms or “heuristic” algorithms. Exact algorithms are those that yield an optimal solution. As discussed in Section 3.1 different exact solution techniques have been used to solve the GAP and in some research, the authors used some optimization programming languages like CPLEX and AMPL.

Basically the GAP is a QAP and it is an NP-hard problem as shown in Obata [21]. Since the AGAP is NP-hard, researchers have suggested various heuristic and metaheuristics approaches for solving the GAP. With heuristic algorithms, theoretically there is a chance to find an optimal solution. That chance can be remote because heuristics often reach a local optimal solution and get stuck at that point. But metaheuristics or “modern heuristics” introduce systematic rules to deal with this problem. The systematic rules avoid local optima or give the ability of moving out of local optima. The common characteristic of these metaheuristics is the use of some mechanisms to avoid local optima. Metaheuristics succeed in leaving the local optimum by temporarily accepting moves that cause worsening of the objective function value. Sections 3.2 and 3.3 addressed the heuristic and metaheuristics approaches for solving the GAP. Some papers presenting good overviews as well as annotated bibliographies on the topic of GAP and a good literature on the AGAP and the use of metaheuristics for AGAP are Dorndorf et al. [20, 22] and Cheng et al. [23].

This paper surveys a large number of models and techniques developed to deal with GAP. In Section 2, we detail the models formulations of the problem. In Section 3, we addressed the resolution methods used to solve the problem. We conclude in Section 4, and we represent the research trends.

## 2. Formulations of AGAP and Related Problems

Many researchers formulated the AGAP as an integer, binary, or mixed integer linear or nonlinear model and some of them formulated it as binary or mixed binary quadratic models, whereas some of the researchers have formulated the AGAP as well-known related problems in combinatorial optimization such as quadratic assignment problem (QAP), clique partitioning problem (CPP), and scheduling problem or even as a network representation. However, some of the researchers formulated the AGAP as a robust optimization model. In this section, according to the way of how the researchers deal with the gate assignment problem, a classification for the AGAP has been made as follows.

### 2.1. Selected AGAP Formulations

#### 2.1.1. Integer Linear Programming Formulations (IP)

Lim et al. [24] formulated the AGAP as an integer programming model and developed two models with time windows. The first model was devoted to minimization of the passenger walking distance (travel time)

(1) $$\begin{array}{c} \text{Minimize}\;\;\overset{n}{\underset{i=1}{\sum }}\;\overset{n}{\underset{j=1}{\sum }}\;\overset{m}{\underset{k=1}{\sum }}\;\overset{m}{\underset{l=1}{\sum }}f_{ij}w_{kl}z_{ijkl}+\overset{n}{\underset{i=1}{\sum }}p_{i}\left(c_{i}-a_{i}\right) \end{array}$$

while the second model optimized the gate assignments with cargo handling costs:

(2) Minimize  ∑i=1n ∑j=1n ∑k=1m ∑l=1mfijAckl+cA+fijBckl+cBzijkl +∑i=1npici−ai.

Both of these objectives put a penalty function due to a delay. These two objectives used the constraints, as follows:

(3)  ∑k=1mxik=1, 1≤i≤n,

(4)  zijkl≤xik, 1≤i,  j≤n,  1≤k,  l≤m,

(5)  zijkl≤xjl, 1≤i,  j≤n,  1≤k,  l≤m,

(6)  xik+xjl−1≤zijkl, 1≤i,  j≤n,  1≤k,  l≤m,

(7)  ci≥ai, 1≤i≤n,

(8)  ci≤bi−di, 1≤i≤n,

(9)  ci+di−ci+yijM>0, 1≤i,  j≤n,

(10)  ci+di−ci−1−yijM≤0, 1≤i,  j≤n,

(11)  yij+yji≤zijkl, 1≤i,  j≤n,  i≠j,  1≤k≤m,

where *x*_*ik*_, *y*_*ij*_, and *z*_*ij* *kl*_ are binary and *c*_*i*_ is integer.

Constraint (3) ensures that each flight must be assigned to exactly one gate. Constraints (4)-(5) state that a binary variable *z*_*ij* *kl*_ can be equal to one if flight *i* is assigned to gate *k* (*x*_*ik*_ = 1) and flight *j* is assigned to gate *l* (*x*_*jl*_ = 1). Constraint (6) further specifies the necessary condition that *z*_*ij* *kl*_ must be equal to one if *x*_*ik*_ = 1 and *x*_*jl*_ = 1. Constraints (7) and (8) ensure that the flight must land and depart within the specified time window. Constraint (9) indicates that *y*_*ij*_ = 1 = 1 if (*c*_*i*_ + *d*_*i*_) ≤ *c*_*j*_, which means *y*_*ij*_ = 1 when flight *i* departs before or right at the time when some gate opens for flight *j*. Constraint (10) states that *y*_*ij*_ = 0 if (*c*_*i*_ + *d*_*i*_) > *c*_*j*_, which means *y*_*ij*_ = 0 when flight *i* departs after some gate opens for flight *j*. Constraint (11) specifies that one gate cannot be occupied by two different flights simultaneously.

In the first model and according to the linearity of the objective function and constraints, they used a standard IP solver (CPLEX) to find the optimal solution, whereas in the second model authors used several heuristic algorithms, namely, the “Insert Move Algorithm,” the “Interval Exchange Move Algorithm,” and a “Greedy Algorithm” to generate solutions. The generated solutions then have been improved using a tabu search (TS) and memetic algorithm. The results showed that the used heuristics performed better than the IP solver (CPLEX) in both CPU time and solutions quality.

Diepen et al. [25, 26] formulated the AGAP as integer linear programming model with a relaxation for the integrality. After relaxing the integrality, the resulting relaxed LP was exploited to obtain solutions of ILP by using column generation (CG). The problem was divided into two phases, planning and attaching. The first phase was the planning section and it was easier to model and calculate. Their objective is to minimize the cost of a gate plan. They proposed the following model:

(12) $$\begin{array}{c} \text{Minimize}\;\overset{n}{\underset{j=1}{\sum }}c_{j}x_{j} \end{array}$$

subject to

(13) $$\begin{array}{c} \overset{n}{\underset{j=1}{\sum }}g_{ij}x_{j}+U_{i}=1\;\text{for}\;i=1,\ldots ,m, \end{array}$$

where

(14)   gij=1,if  flight  i  is  in  gate  plan  j,0,otherwise, Ui≥0 for  i=1,…,m,  Ui  is  a  penalty  variable.

This constraint defined the high price penalty (*U*_*i*_) when the flights were not assigned to the gates. This penalty appeared since the planner should do the assignment manually. In addition, they added another constraint regarding the assignment since there was possibility that a long stay flight could be split into two parts. The extra flights *i*_*a*_ and *i*_*b*_ that refer to the arrival and departure of flight *i* were added to the previous constraints:


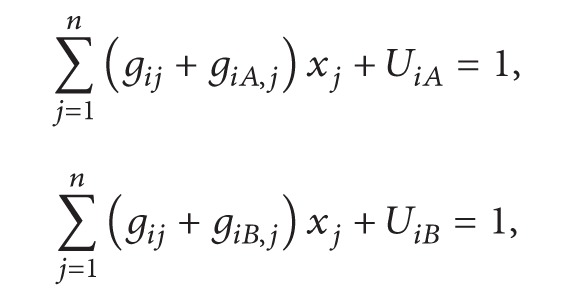

(15)



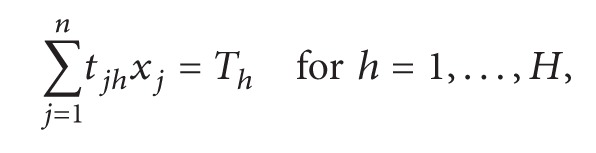

(16)



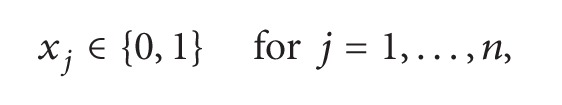

(17)

where


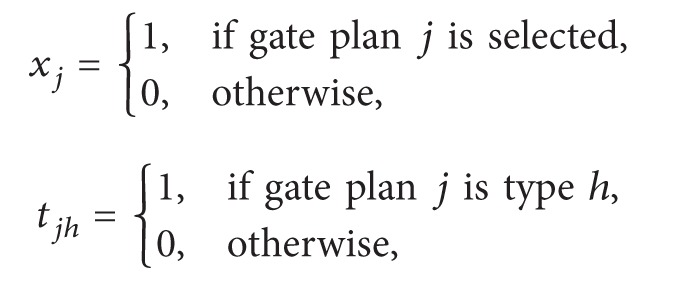

(18)



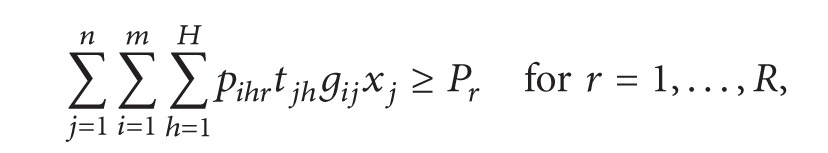

(19)

where

(20) pihr =1,if  flight  i  has  preference  on⁡  gate  type  hin  preference  r,0.5,if  the  unsplit  version  of  flight  i  has  preference  on⁡  gate  type  h  in  preference  r,0,otherwise,

and *R* denotes the total number of preferences.

This constraint defined the flight preferences; for example, a flight should be assigned to the same gate due to the ownership or security. The coefficient 0.5 refers to the extra flight defined in constraint (15).

They checked the solution's optimality using pricing problem (minimum reduced cost) since they had dual multipliers *π*_*i*_, *λ*_*h*_, and *ψ*_*r*_ for constraints (15), (16), and (19), respectively:

(21) $$\begin{array}{c} c_{j}-\overset{H}{\underset{h=1}{\sum }}t_{jh}\lambda_{h}-\overset{m}{\underset{i=1}{\sum }}\left(g_{ij}\pi_{i}+\overset{R}{\underset{r=1}{\sum }}\;\overset{H}{\underset{h=1}{\sum }}\left(g_{ij}t_{jh}p_{ihr}\psi_{r}\right)\right). \end{array}$$

The second phase was a matter of assignment in physical gate. They made the rules to solve this phase as follows.

- Sort the gates based upon the quality.
- Sort the gate plans from the highest on the total number of departing passengers that are on the flights in that gate plan.
- Assign the gate plan to the best gate considering the highest number of departing passengers, assign the next gate plan to the next-best gate, and so on.

In [26], Diepen et al. used the solution obtained from their assignment of gates as an input to solve the bus-planning problem in the same airport.

#### 2.1.2. Binary Integer Programming

In 2009, Tang et al. [27] formulated the AGAP as a binary integer programing model as below. The output model was used to generate a lower bound to their original problem:

(22) $$\begin{array}{c} \text{Minimize}\;Z=\underset{i\in I}{\sum }\;\underset{j\in E_{i}}{\sum }\;\underset{k\in D_{ij}}{\sum }d_{ik}x_{ijk}+\;\underset{i\in I}{\sum }\;\underset{j\in E_{i}}{\sum }\;\underset{k\in D_{ij}}{\sum }w_{ij}x_{ijk} \end{array}$$

subject to

(23)  ∑j∈Ei ∑k=Dijxijk  =1, ∀i∈I,

(24)  ∑i∈Fjs ∑k=Hisxijk  ≤1, ∀j∈G,  ∀s∈S,

(25)  ∑i∈Ltq ∑j∈Nq ∑k∈Hisxijk≤1, ∀t∈Tq,  ∀q∈Q,  ∀s∈S,

(26) xijk=1,if  flight  i  is  assign  to  gate  j  at  the  time  point  (starting  point)  k0,otherwisegggggghhhgggg∀k∈Dij, ∀j∈Ei, ∀i∈I.

*Parameter Variables*

*d* _*ik*_ : time inconsistency value indicating that the *i*th flight is assigned at the *k*th time point (starting time); if *k* is equal to the original time point, then *d*_*ik*_ = 0;
*w* _*ij*_ : space inconsistency value indicating that the *i*th flight is assigned to the *j*th gate; if *j* equals the original gate, then *w*_*ij*_ = 0.

The following sets have been defined:

*I*: considered flights;
*G*: available gates;
*E* _*i* _: gates that the *i*th flight can be assigned to;
*D* _*ij* _: time points in which the *i*th flight can be assigned to the *j*th gate;
*F* _*js* _: flights that can be assigned to the *j*th gate so that their time windows will cover the *s*th time point;
*S*: all time points (i.e., the time points from the planning time at each stage to the end of daily operations);
*H* _*is* _: time points (starting times) assigned to the *i*th flight, where the resulting time windows cover the *s*th time point;
*T* _*q* _: conflicting flight pairs for the *g*th adjacent gate pair;
*L* _*tq* _: flights included in the *t*th conflicting flight pair for the *q*th adjacent gate pair;
*Q*: adjacent gate pairs.

Equation (23) is the flight constraint, indicating that every flight is exactly assigned to a gate. Equation (24) is the gate constraint, ensuring that every gate is assigned to at most one flight at any time. Constraint (25) is related gate adjacency, denoting that two conflicting flights cannot be concurrently assigned to an adjacent gate pair. Constraint (26) indicates that the assignment variables are either zero or one.

Kumar et al. [18] presented a binary integer programing model that produced a feasible gate plan in the light of all the business constraints:

(27)  xik=1,if  turn  i  is  assigned  to  gate  k,0,otherwise, yi=1,if  turn  i  is  not  assigned  to  any  gate,0,otherwise, wi=1,if  long  turn  t  is  towed,0,otherwise, Maximize  ∑i∈T ∑k∈KCikxik−C1∑t∈Lwt−C2∑i∈Tyi

subject to

(28)  ∑k∈Kxik+yi=1, i∈T,      

(29) $$\begin{array}{c} \;\underset{k\in K;e_{i}\in E_{k}}{\sum }x_{ik}+y_{i}=1,\;i\in T,\; \end{array}$$

(30)  ∑i∈Tyi≤ξ,

(31) yik+yjk≤1, i,j∈T;  k∈K:ai<bj+α,fffffffffjjggfffffaj<bi+α,  i≠j

(32) yik+yjl≤1, i,j∈T;  l∈K;gggk,l∈J:ai<bj,  aj<bi,fffHfi≠j,  ei∈Ek1,  ej∈El1

(33) yik+yjl≤1, i,j∈T; k,l∈K;kLiFoF,kLiFoR∈LF:aj≤ai≤bj,hhi≠j, ei∈EkLiFoF, ej∈ElLiFoR

(34) yik+yjl≤1, i,j∈T;  k,l∈K; kLiFoF,lLiFoR∈LF:aj≤bi≤bj, i≠j, ei∈EkLiFoF, ej∈ElLiFoR

(35) yik+yjl≤1,   i,j∈T;  k,l∈K;kPB,lPB∈JPB:bi−β<bj≤bi+β,ggggggggggi≠j, ei∈Ek, ej∈El

(36)  wt≤τ,   t∈TL    

(37) yi1k−yi2k≤wt, i1,i2∈T,  t∈TL,fffk∈K:i1≠i2, pi1=pi2=t

(38) yi1k−yi3k−1≤wt, i1,i2,i3∈T,  t∈TL,k∈K:ai1<ai3, bi3<ai2, pi1=l, pi2=l

(39) yi1k1−yi3k2−1≤wt, i1,i2,i3∈T,  t∈TL,k1,k2∈K; j∈J:ai1<ai3, bi3<bi2, pi1=l,pi2=l, k1=qj1, k2=qj2, ei1∈Ej1, ei2∈Ej2.

Constraint (28) ensures that turn *i* is assigned to at most one gate. Constraint (29) states that turn *i* is assigned to a gate only if its equipment type is among the types which the assigned gate can accommodate. Constraint (30) restricts the number of ungated turns to less than or equal to the allowed number *ξ*. Constraint (31) shows that, at any given time, at most one turn is assigned to one gate. Constraint (32) ensures that adjacency constraints are observed. Constraints (33)-(34) enforce LIFO restrictions. Constraint (35) guarantees that pushback restrictions are observed. Constraint (36) confirms that no turn is towed if towing is not allowed. Finally, constraints (37)–(39) certify that if a long turn *t* is towed, the *w*_*t*_ variable is set to be 1.

Mangoubi and Mathaisel [11] also developed a binary integer model to minimize the passenger total walking distance and proposed a heuristic method to find the solution. The heuristic method result has been compared with the results from a standard IP solver and the comparison results showed that the heuristic method was superior to the LP solver; the average walking distance using the LP is 527 feet while heuristic is 558 feet. The developed model is introduced as follows:

(40) $$\begin{array}{c} \text{Minimize}\;Z=\overset{M}{\underset{i=1}{\sum }}\;\overset{M}{\underset{i=1}{\sum }}\left(p_{i}^{a}d_{j}^{a}+p_{i}^{d}d_{j}^{d}+p_{i}^{t}d_{j}^{t}\right)x_{ij}, \end{array}$$

where

(41) $$\begin{array}{c} x_{ij}=\left\{\begin{array}{cc} 1, & \text{if}\;\text{flight}\;i\;\text{is}\;\text{assigned}\;\text{to}\;\text{gate}\;j,\\ 0, & \text{otherwise}. \end{array}\right. \end{array}$$

Transfer passenger walking distances are determined from a uniform probability distribution of all intergate walking distances. The expected walking distance if *w*_*jk*_ is the distance between gate *j* and gate *k* is

(42) $$\begin{array}{c} d_{j}^{t}=\frac{1}{N}\overset{N}{\underset{k=1}{\sum }}w_{jk}\;\forall j=1,\ldots ,N \end{array}$$

subject to

(43)  ∑j=1Nxij=1 ∀i=1,…,M

(44)  ∑h∈Lixhj+xij≤1 ∀i=1,…,M,  ∀j=1,…,N,

(45) ∑h∈L(p+1)xhj+xp+1,j=xp−3,j+xp−2,j   +xp−1,j+xpj+xp+1,j≤1,

(46)  Lp⊂Lp+1⊂⋯⊂Lp+k,

(47)  ∑z=1N ∑s=1Nxgzwzsxhs≤Dghmax⁡,

(48)  pfadsa+pfddsd+pftdst=min⁡j∈S⁡pfadja+pfddjd+pftdjt.

Constraint (43) shows that each flight is assigned to at most one gate. Constraint (44) ensures that no two planes are assigned to the same gate concurrently. Constraint (45) determines the conflict constraint for each gate *j*. Constraint (46) is written to consider only the constraint generated by the last plane of two or more flights arriving with no departure in between. Constraint (47) ensures that flights are assigned to nearby gates. Constraint (48) assigns flight *f* to gate *s* ∈ *S*, where *s* is the gate with the minimum total passenger walking distance for flight *f*.

Vanderstraeten and Bergeron [28] formulated the GAP as a binary integer model but with the objective of minimizing the off-gate events and they developed a new heuristic, which is the “Affectation Directe des Avions aux Portes (ADAP),” to solve the developed model. A real case has been studied in an Air Canada terminal. A new heuristic was applied to real data at Toronto International Airport. The developed model was as follows:

(49) $$\begin{array}{c} \text{Maximize}\;Z=\underset{i\in I}{\sum }\;\underset{j\in J}{\sum }V_{ij} \end{array}$$

subject to

(50)  ∑j∈JiVij≤1, i∈I,

(51)  Vij+∑k∈AijVkj≤1, i∈I,  j∈Ji,

(52)  Vij+∑k∈BijrVkr+∑k∈BijlVkl≤1, i∈I,  j∈Ji,

(53)  Vij=0,1 i∈I,  j∈Ji.

Constraint (50) ensures that each flight is assigned to at most one gate. Constraint (51) defines the occupation time at any gate. Constraint (52) determines the neighboring constraint. Constraint (53) expresses the binary constraint for all decision variables. The results showed that using the developed method resulted in no more than 30 events ever being handled off gate while the manual procedure obtained events up to 50 of the 300 events being handled off gate.

Bihr [12] developed a binary integer model to minimize the passenger walking distance and applied this model to solve a sample problem using primal-dual simplex algorithm. As a result, he obtained a total walking distance of 22,640. The developed model is introduced as follows:

(54) $$\begin{array}{c} \text{Minimize}\;\theta =\sum C_{ij}X_{ij},\;i=1,\ldots ,m,\;\;j=1,\ldots ,k, \end{array}$$

where the *C*_*ij*_ are the elements of the matrix product of PAX(*i*, *j*)∗DIST(*i*, *j*)^*T*^ and

 PAX(*i*, *j*) = number of passengers arriving on flight *i* and departing from gate *j*;
 DIST(*i*, *j*) = number of passengers − distance units from gate *i* to gate *j*;
 
*X*_*ij*_ = 0 or 1

subject to

(55) $$\begin{array}{c} \sum X_{ij}=1,\\ \sum X_{ji}=1. \end{array}$$

In 2002, Yan et al. [29] formulated the static GAP as a binary integer programing model to serve as a basis of real time gate assignments in a simulation framework developed to analyze the effects of stochastic flight delays on static gate assignments. The presented model is as follows:

(56) $$\begin{array}{c} \text{Minimize}\;Z=\overset{M}{\underset{i=1}{\sum }}\;\overset{N}{\underset{j=1}{\sum }}c_{ij}x_{ij} \end{array}$$

subject to

(57)  ∑j=1Nxij=1 ∀i,

(58)   ∑i∈Lsxij≤1 ∀s,  ∀j,

(59)  xij=0  or  1 ∀i,  ∀j.

Constraint (57) ensures that each flight is assigned to at most one gate. Constraint (58) certifies that no two planes are assigned to the same gate concurrently. Constraint (59) is related to the binary constraint for all decision variables. Two greedy heuristics were used to solve the model and their results were compared with the insights of the optimization method. The simulation framework was tested to solve certain real case instances from CKS airport. The results of the used methods were 24,562,588 for the optimization model and 27,833,552 and 30,166,809 (meters) for the two greedy heuristics.

#### 2.1.3. Mixed Integer Linear Programming (MILP)

Bolat [30] formulated a mixed integer program for the AGAP with the objective of minimizing the range of slack times (slack time is an idle time between two successive utilizations of the gate). Certain instances, with more than 20 gates, have been considered according to airplane types, gate types, terminal types, and utilization levels:

(60) $$\begin{array}{c} \text{Minimize}\;v^{+}\left(\Gamma \right)-v^{-}\left(\Gamma \right) \end{array}$$

subject to

(61)  v+Γ=max⁡viΓ, i=1,…,N+M,

(62)  v−Γ=min⁡viΓ, i=1,…,N+M,

(63)  viΓ=Ai−Ei,Γi, i=1,…,N,

(64) Ei,Γi=Dk∗,where  k∗=max⁡k,  Γk=Γi,  k=1,…,i−1,BΓ,if  no  such  k  exist,

(65)  vN+jΓ=Tj−EN+1,j, j=1,…,M.

The results related to expected average utilizations were, respectively, 88.54%, 67.13%, and 45.57% over heavily utilized, normally utilized, and underutilized problems. Concerning the average number of flights, results were 10%, 7.59%, and 5.15% per gate.

In 2001, Bolat [31] presented a framework for the GAP that transformed the nonlinear binary models (it will be discussed in Section 2.1.4 according to our classification) into an equivalent linear binary model with the objective of minimizing the range or the variance of the idle times. The framework consists of five mathematical models, where two of the five models were formulated as a mixed integer linear programming and the others as a mixed integer nonlinear programming. Models P1 to P4 were defined for homogenous gate while model P5 was defined for heterogeneous gate:

(66)  Xjk=1,if  flight  j  is  assigned  to  gate  k,0,otherwise, Pjk=1,if  the  assignment  of  flight  j  to  gate  k  cansatisfy  all  considerations,0,otherwise.

Using the presented framework, nonlinear model P1 (model P1 will be discussed in Section 2.1.4 according to our classification) was transformed to the following mixed integer linear model, which is model P2.

*Model P2*. Consider

(67) $$\begin{array}{c} \text{Minimize}\;S_{\max}-S_{\min} \end{array}$$

subject to

(68)   Smax⁡≥Sjk j=1,…,N,  k=1,…,M,

(69)   Smin⁡≤Sjk+1−XjkZ j=1,…,N,  k=1,…,M,

(70)    Smax⁡,Smin⁡≥0,

(71)    ∑k=1MPjkXjk=1 j=1,…,N,

(72a)   E1k=MaximizeA1X1k,Bk k=1,…,M,

(72b) Ejk=MaximizeAjXjk,Lj−1,k j=2,…,N,hhhhhhhhhhhhhhihhhhhhhhhk=1,…,M,

(73)   Ljk=Ejk+GjXjk   j=1,…,N,  k=1,…,M,

(74a)   S1k=E1k−Bk k=1,…,M,

(74b) Sjk=Ejk−Lj−1,k j=2,…,N,hhhhhhhhhhhh k=1,…,M,

(75) $$\begin{array}{c} S_{N+1,k}=F_{k}-L_{Nk}\;k=1,\ldots ,M, \end{array}$$

(76)   Xjk=0  or  1 j=1,…,N,  k=1,…,M,

(77)   Ejk,Ljk,Sjk,SN+1,k≥0 j=1,…,N,  k=1,…,M.

Similarly, for model P3 (Section 2.1.4), the resultant model was model P4 that is a mixed binary model as in model P2, but with two additional real variables as follows.

*Model P4*. Consider

(78) $$\begin{array}{c} \text{Minimize}\;S_{\max}-S_{\min} \end{array}$$

subject to

(79)   Smax⁡≥IijYij i=0,…,N,  j=i+1,…,N+1,

(80)   Smin⁡≤IijYij i=0,…,N,    j=i+1,…,N+1,

(81)  Smax⁡,Smin⁡≥0,

(82)  ∑i=0N ∑j=i+1N+1Yij=N+M,

(83)  ∑j=1N+1Y0j≤M,

(84)  ∑i=0NYi,N+1≤M,

(85)   ∑i=0j−1Yij=1 j=1,…,N,

(86)  ∑j=i+1N+1Yij=1 i=1,…,N,

(87)  Yij=0  or  1 i=0,1,…,N,  j=i+1,…,N+1.

Different instances have been studied according to the number of the gates: small (five gates), medium (10 gates), and large (20 gates). Instances with more than 20 gates were not considered. The results were as follows: average numbers of flight were 26.125, 52.25, and 105.417 and the average utilizations were 45.725, 66.548, and 88.871% according to the gate size, respectively.

Şeker and Noyan [9] formulate the GAP as a mixed integer program with the objective of minimizing the number of conflicts and at the same time minimizing the total semideviation between idle time and buffer time:

(88) $$\begin{array}{c} \text{Minimize}\underset{i\in N^{c}\setminus \left\{0\right\}}{\sum }\;\underset{s\in S}{\sum }R_{i,s}p_{s}+\Lambda \underset{i\in N^{c}}{\sum }\;\;\underset{j\in N^{c}}{\sum }\;\;\underset{s\in S}{\sum }c_{i,j,s}p_{s}. \end{array}$$

Another model was developed as a mixed integer program for the same objective function. The model was the same as the previous model but with some differences:

(89) $$\begin{array}{c} \text{Minimize}\underset{i\in N^{c}\setminus \left\{0\right\}}{\sum }\;\underset{s\in S}{\sum }H_{i,s}p_{s}+\Lambda \underset{i\in N^{c}}{\sum }\;\underset{j\in N^{c}}{\sum }\;\underset{s\in S}{\sum }c_{i,j,s}p_{s}, \end{array}$$

where

(90) $$\begin{array}{c} H_{i,s}=\left\{\begin{array}{cc} 1, & \text{if}\;\text{idle}\;\text{time}\;\text{of}\;\text{flight}\;i\;\text{is}\;\\ & \text{less}\;\text{than}\;\text{the}\;\text{buffer}\;\text{time}\\ 0, & \text{otherwise}. \end{array}\right. \end{array}$$

These two models have the same constraints properties,

(91)  ∑k∈Mxi,k=1, i∈N,

(92)  x0,k=1, k∈M,

(93)  xn+k,k=1, k∈M,

(94)  ∑j∈Li,sxj,k+xi,k≤1, i∈Nc,  k∈M,  s=0,

(95)  ci,j,s≥xi,k+xj,k−1, i∈Nc,  k∈M,  s∈S,  j∈Li,s,

(96)  Ri,s≥b−Ii,s, i∈Nc∖0,  s∈S,

(97) Di,s≥xi,k+xj,k−2Z+dj,s, i∈Nc∖0,  k∈M,hhhhhhhhhhhhgghhhhhhhhhhhhjjhhhhs∈S,j∈Qi,s

(98)  Ii,s≤ai,s−Di,s+b∑j∈Ncci,j,s, i∈Nc∖0,  s∈S,

(99)  xi,k∈0, i∈Nc,  k∈M,

(100)  All  remaining  variables≥0,

while objective (89) has the following additional constraints:

(101) $$\begin{array}{c} H_{i,s}b\geq b-I_{i,s},\;i\in N^{c}\setminus \left\{0\right\},\;\;s\in S, \end{array}$$

(102) $$\begin{array}{c} H_{i,s}\in \left\{0,1\right\},\;i\in N^{c}\setminus \left\{0\right\},\;\;s\in S. \end{array}$$

#### 2.1.4. Mixed Integer Nonlinear Programming

Li [5] formulated the GAP as a nonlinear binary mixed integer model hybrid with a constraint programing in order to minimize the number of gate conflicts of any two adjacent aircrafts assigned to the same gate. The developed model has been solved using CPLEX software:

(103) $$\begin{array}{c} \text{Minimize}\;\underset{i\in N}{\sum }\;\underset{j<i,j\in N}{\sum }y_{i,j}*E\left(p\left(i,j\right)\right), \end{array}$$

where

(104) $$\begin{array}{c} E\left(p\left(i,j\right)\right)=\frac{1}{a_{i}-d_{j}+2b}, \end{array}$$

where *a*: scheduled arriving time, *d*: scheduled departure time, and *b*: buffer time (constant). Consider

(105)  xi,k=1,if  and  only  if  aircraft  fi  is  assigned  to  gate  ck,0,otherwise  1≤i≤n,1≤k≤c, yi,j=1,if  ∃k,xi,k=xj,k=1  1≤k≤c,0,otherwise  1≤i,j≤n.

In another work, Li [32] defined the objective as

(106) $$\begin{array}{c} \text{Minimize}\underset{i,j\in N;i\neq j}{\sum }\frac{y_{i,j}}{a_{i}-d_{j}+2b}. \end{array}$$

These two models have the same constraints; all constraints are as follows.

(107)  ∑i∈N ∑k∈Cxi,k=1,

(108)   ∑i∈N ∑j<i,j∈N ∑k∈Cxi,k∗xj,k=yi,j,

(109)  yi,k∗yj,k∗di−aj∗dj−ai≤0,

(110)  xi,k∈0,1,

(111)  ∀1≤i, j≤n, i≠j, ∀1≤k≤c.

Constraint (107) indicates that each aircraft is assigned to at most only one gate. Constraint (108) represents a method to compute the auxiliary variable *y*_*ij*_ from *x*_*ik*_. Constraint (109) ensures that one gate can only be assigned at most one aircraft at the same time. Some additional constraints in the real operations are ignored. Constraint (110) represents binary value of the decision variables.

As mentioned in Section 2.1.3, Bolat [31] proposed two models formulated as a mixed integer linear program which have been transformed from a mixed integer nonlinear program. The proposed mixed integer nonlinear program was as follows:

(112)  Xjk=1,if  flight  j  is  assigned  to  gate  k,0,otherwise, Pjk=1,if  the  assignment  of  flight  j  to  gate  kcan  satisfy  all  considerations,0,otherwise.

*Model P1.* Consider

(113) $$\begin{array}{c} \text{Minimize}\;\overset{M}{\underset{k=1}{\sum }}\overset{N+1}{\underset{j=1}{\sum }}S_{jk}^{2} \end{array}$$

subject to

(114) $$\begin{array}{c} \;\overset{M}{\underset{k=1}{\sum }}P_{jk}X_{jk}=1\;j=1,\ldots ,N, \end{array}$$

(115a)   E1k=MaximizeA1X1k,Bk k=1,…,M,

(115b) Ejk=MaximizeAjXjk,Lj−1,k j=2,…,N,hhhhhhhhhhhhggghhhhhhhhhhhk=1,…,M,

(116)   Ljk=Ejk+GjXjk j=1,…,N,  k=1,…,M,

(117a)   S1k=E1k−Bk k=1,…,M,

(117b)   Sjk=Ejk−Lj−1,k   j=2,…,N,  k=1,…,M,

(118)   SN+1,k=Fk−LNk k=1,…,M,

(119)   Xjk=0  or  1 j=1,…,N,  k=1,…,M,

(120)   Ejk,Ljk,Sjk,SN+1,k≥0 j=1,…,N,  k=1,…,M.

Bolat [31] also proposed two alternative formulations for homogenous and heterogeneous gates. The proposed extended formulation for the homogenous gates was as follows:

(121) $$\begin{array}{c} \;Z=\underset{k=1,\ldots ,M}{\max}\left\{F_{k}-B_{k}\right\}, \end{array}$$

(122) $$\begin{array}{c} \;I_{ij}=\left\{\begin{array}{cc} A_{j}-D_{i}, & \text{if}\;A_{j}\geq D_{i},\\ Z, & \text{otherwise}, \end{array}\right. \end{array}$$

(123) $$\begin{array}{c} \;I_{0j}=A_{j}\;j=1,\ldots ,N, \end{array}$$

(124) $$\begin{array}{c} \;I_{i,N+1}=H-D_{i}\;i=1,\ldots ,N. \end{array}$$

*Model P3*. Consider

(125) $$\begin{array}{c} \text{Minimize}\;\overset{N}{\underset{i=0}{\sum }}\;\overset{N+1}{\underset{j=i+1}{\sum }}I_{ij}^{2}Y_{ij} \end{array}$$

subject to

(126)   ∑i=0N ∑j=i+1N+1Yij=N+M,

(127)   ∑j=1N+1Y0j≤M,

(128)   ∑i=0NYi,N+1≤M,

(129)   ∑i=0j−1Yij=1 j=1,…,N,

(130)   ∑j=i+1N+1Yij=1 i=1,…,N,

(131)   Yij=0  or  1 i=0,1,…,N,  j=i+1,…,N+1.

In addition, the proposed extended formulation for the heterogeneous gates was as follows:

(132) Iijk=Aj−Di,if  Ai>Bk,  Aj≥Di,ghhgggPik=Pjk=1,Z,otherwise,

(133)   I0jk=Aj−Bk,if  Aj≥Bk,Z,otherwise,

(134)   Ii,N+1,k=Fk−Di,if  Di≤Fk,Z,otherwise.

*Model P5.* Consider

(135) $$\begin{array}{c} \text{Minimize}\;\overset{M}{\underset{k=1}{\sum }}\;\overset{N}{\underset{i=0}{\sum }}\;\overset{N+1}{\underset{j=i+1}{\sum }}I_{ijk}^{2}Y_{ijk} \end{array}$$

subject to

(136)  ∑k=1M ∑i=0N ∑j=i+1N+1Yijk=N+M,

(137)  ∑j=1N+1Y0jk≤1 k=1,…,M,

(138)  ∑k=1M ∑i=0j−1Yijk=1 j=1,…,N,

(139)  ∑k=1M ∑j=i+1N+1Yijk=1 j=1,…,N,

(140) Yijk+∑t=1t≠kM ∑v=j+1N+1Yjvt≤1 i=0,…,N−1fffffffffj=i+1,…,N  k=1,…,M,

(141) Yijk=0  or  1 i=0,…,N,  j=i+1,…,N+1,hhhhhhhhhhhhhhhhhohhhhhhk=1,…,M

(142)  Yabc+∑t=1t≠cM ∑v=b+1N+1Ybvt≤1.

As mentioned in Section 2.1.3, different instances have been studied according to the number of the gates: small (five gates), medium (10 gates), and large (20 gates). Instances with more than 20 gates were not considered. The results were as follows: average numbers of flight were 26.125, 52.25, and 105.417 and the average utilizations were 45.725, 66.548, and 88.871% according to the gate size, respectively.

#### 2.1.5. Quadratic Programming

(*1) Quadratic Mixed Binary Programming.* Zheng et al. [33] formulated the GAP as a mixed binary quadratic program with minimizing the slack time overall variance as the objective function; an assumption has been stated such that the flights are sequenced with the smallest arrival time. The proposed mixed binary quadratic model was as follows:

(143) $$\begin{array}{c} \text{Minimize}\;f=\overset{N+1}{\underset{i=1}{\sum }}\;\overset{M}{\underset{k=1}{\sum }}{\left(R_{ik}-\overset{-}{R}\right)}^{2} \end{array}$$

subject to

(144)  ∑k∈Myik=1,

(145)  yik≥∑j∈Nzijk,

(146)  yjk≥∑i∈Nzijk,

(147)  Rjk=aj−STk, j=min⁡⁡j,  j∈j ∣ yjk=1,

(148)  RN+1,k=ETk−di, i=max⁡⁡i,  i∈i ∣ yjk=1,

(149)  Rik=aj−di, ∀i,j,k∈i,j,k ∣ zijk=1,

(150)  aj+1−zijkS≥di+I,

(151)  vi≤ek+1−yikS,

(152)  yik=1,if  flight  i  is  assigned  to  gate  k,0,otherwise,

(153)  zijk=1,if  flight  i  and  flight  j  are  both  assigned  to  gate  k,0,otherwise,

where the indices *i*, *j*, *k* in (143)–(151) denote *i*, *j* ∈ *N*, *k* ∈ *M*. Equation (143) represents the objective function with the aim of minimizing overall variance of slack time. Constraint (144) imposes the assignment of every flight to one gate. Constraint (145) obliges every flight to have at most one immediate precedent flight. Constraint (146) enforces every flight to have at most one immediate succeeding flight. Constraints (147) and (148) define the first and last slack time of each gate, and constraint (149) defines the other slack times. Constraint (150) stipulates that the flight can be assigned to the gate when the preceding flight has departed for dwell time. Constraint (151) indicates that the different type of gate allows parking different type of flight.

Solutions were obtained using tabu search based on some initial (starting) solutions; the results were compared with those of a random algorithm developed in the literature. Using data from Beijing International Airport (10 gates and 100 of flights between 6:00 and 16:00), the initial solutions using metaheuristic and random algorithm were 9821 and 15775, respectively.

Bolat [34] formulated the AGAP as a mixed binary quadratic programming model to minimize the variance of idle times and used branch and bound algorithm and proposed two heuristics which were “single pass heuristic” (SPH) and “heuristic branch and bound” (HBB) for solving the proposed model. The proposed mixed binary quadratic model was stated as follows:

(154) $$\begin{array}{c} \text{minimize}\;Z=\overset{M}{\underset{k=1}{\sum }}\;\overset{N+1}{\underset{j=1}{\sum }}S_{jk}^{2} \end{array}$$

subject to

(155)  ∑k=1MPjkXjk=1, j=1,…,N,

(156)  Ejk≥AjXjk, j=1,…,N,  k=1,…,M,

(157)  Ejk≥Lj−1,k, j=1,…,N,  k=1,…,M,

(158)  Ljk=Ejk+GjXjk, j=1,…,N,  k=1,…,M,

(159)  Sjk=Ejk−Lj−1,k, j=1,…,N,  k=1,…,M,

(160)  SN+1,k=LN+1,k−LNk, k=1,…,M,

(161) Xjk=1,if  flight  j  is  assigned  to  gate  k0,otherwise,hhhhhhhhhj=1,…,N, k=1,…,M,

(162) Ejk,Ljk,Sjk,SN+1,k≥0, j=1,…,N,hhhhhhhhhhhhhhhhhhhhk=1,…,M.

Real instances, from King Khalid International Airport (72 generated sets), were used. During the initial phase, the proposed heuristic methods gave an average improvement of 87.39% on the number of remote assigned flights, whereas the average improvement on the number of towed aircrafts during the real time phase was 76.19%.

Xu and Bailey [14] formulated the GAP as a mixed binary quadratic programming model (Model 1) and the objective was to minimize the passenger connection time. The proposed model (Model 1) was reformulated (linearized) into another model (Model 2) in which the objective function and the constraints have been linearized (the resultant model was a mixed binary integer model). Model 1 and Model 2 are listed below.

*Model 1.* Consider

(163) $$\begin{array}{c} \text{Minimize}\;\underset{i,j\in N}{\sum }\;\underset{k,l\in K}{\sum }f_{ij}c_{kl}y_{ik}y_{jl} \end{array}$$

subject to

(164)  ∑k∈Kyik=1, ∀i∈N,

(165)  yik=∑j∈Nzijk, ∀i∈N,  ∀k∈K,

(166)  yjk=∑i∈Nzijk, ∀j∈N,  ∀k∈K,

(167)  ti≥ai  +∝, ∀i∈N,

(168)  ti≤di−θi∑j∈Nfji, ∀i∈N,

(169)  ti+θi∑j∈Nfji≤tj+1−zijkM, ∀i,j∈N,  ∀k∈K,

(170)  aj+1−zijkM≥di+β, ∀i,j∈N,  ∀k∈K,

(171)  yik=1,if  flight  i  is  assigned  to  gate  k,0,otherwise,

(172)  zijk=1,if  flight  i,j  are  both  assigned  to  gate  k;flight  i  immediately  precedes  flight  j,0,otherwise,

(173)  zijk,zijk∈0,1, ∀i,j∈N,  ∀k,l∈K,

(174)  ti≥0, ∀i∈N,

where objective function (163) seeks to minimize the total connection times by passengers. Constraint (164) specifies that every flight must be assigned to one gate. Constraint (165) indicates that every flight can have at most one flight immediately followed at the same gate. Constraint (166) indicates that every flight can have at most one preceding flight at the same gate. Constraints (167) and (168) stipulate that a gate must open for boarding on a flight during the time between its arrival and departure and also must allow sufficient time for handling the passenger boarding, which is assumed to be proportional to the number of passengers going on board. Constraint (169) establishes the precedence relationship for the binary variable *z*_*ij* *k*_ and the time variables *t*_*i*_ and *t*_*j*_ and is only effective when *z*_*ij* *k*_ = 1. It stipulates that if flight *i* is assigned immediately before flight *j* to the same gate *k*, the gate must open for flight *i* earlier than for flight *j*. Therefore, it ensures each gate only serves one flight at any particular time. Constraint (170) further states that the aircraft can only arrive at the gate when the previous flight has departed for certain time.

*Model 2.* Consider

(175) xijkl=1,  if  and  only  if  flight  i  is  assigned  to  gate  k,  and  flight  j  is  assigned  to  gate  l,0,otherwise,

(176) $$\begin{array}{c} \text{Minimize}\;\underset{i,j\in N}{\sum }\;\underset{k,l\in K}{\sum }f_{ij}c_{kl}x_{ijkl} \end{array}$$

subject to

(177)  ∑k∈Kyik=1, ∀i∈N,

(178)  yik=∑j∈Nzijk, ∀i∈N,  ∀k∈K,

(179)  yjk=∑i∈Nzijk, ∀j∈N,  ∀k∈K,

(180)  ti≥ai  +∝, ∀i∈N,

(181)  ti≤di−θi∑j∈Nfji, ∀i∈N,

(182)  ti+θi∑j∈Nfji≤tj+1−zijkM, ∀i,j∈N,  ∀k∈K,

(183)  aj+1−zijkM≥di+β, ∀i,j∈N,  ∀k∈K,

(184)  zijk,zijk∈0,1, ∀i,j∈N,  ∀k,l∈K,

(185)  ti≥0, ∀i∈N,

(186)  xijkl≤yik, ∀i,j∈N,  ∀k,l∈K,

(187)  xijkl≤yjl, ∀i,j∈N,  ∀k,l∈K,

(188)  yik+yjl−1≤xijkl, ∀i,j∈N,  ∀k,l∈K,

(189)  xijkl∈0,1, ∀i,j∈N,  ∀k,l∈K,

where constraints (186) and (187) state that a binary variable *x*_*ij* *kl*_ can be equal to one if flight *i* is assigned to gate *k* (*y*_*ik*_ = 1) and flight *j* is assigned to gate 1 (*y*_*jl*_ = 1). Constraint (188) further gives the necessary condition which is that *x*_*ij* *kl*_ must be equal to one if *y*_*ik*_ = 1 and *y*_*jl*_ = 1.

The B&B and tabu search algorithm were used to solve the generated instances (seven instances, up to 400 flights and 50 gates for 5 consecutive working days). The results of the analyzed instances showed an average saving of the connection time of 24.7%.

(*2) Binary Quadratic Programming.* Ding et al. [6, 35] developed a binary quadratic programming model for the overconstrained AGAP to minimize the number of ungated flights. A greedy algorithm was designed to obtain an initial solution, which has been improved using tabu search (TS). The developed model was stated as follows:

(190)  Minimize  ∑m+1nyi,m+1,

(191) Minimize  ∑i=1n ∑j=1n ∑k=1m+1 ∑l=1m+1fi,jwk,lyi,kyj,l+∑i=1nf0,iw0,i gggggggg+∑i=1nfi,0wi,0

subject to

(192)  ∑k=1m+1yi,k=1 ∀i,  1≤i≤n,

(193)  ai<di ∀i,  1≤i≤n,

(194) yi,kyj,kdj−aidi−aj≤0∀i,  1≤i,  j≤n,  k≠m+1,

(195)  yik=1,if  flight  i  is  assigned  to  gate  k,(0<k≤m+1)0,otherwise

(196)  ∀i,  1≤i≤n,∀k,  1≤k≤m+1,

where constraint (192) ensures that every flight must be assigned to one and only one gate or assigned to the apron. Constraint (193) specifies that the departure time of each flight is later than its arrival time. Constraint (194) says that an assigned gate cannot admit overlapping the schedule of two flights.

In 2005, Ding et al. [7] developed a binary quadratic programming model for the overconstrained AGAP to minimize the number of ungated flights. The developed model was as follows:

(197)  Minimize  ∑m+1nyi,m+1,

(198)  Minimize  ∑i=1n ∑j=1n ∑i=1m+1fi,jwk,lyi,kyj,l+∑i=1nf0,iw0,i+∑i=1nfi,0wi,0

subject to

(199)  ∑k=1m+1yi,k=1 ∀i,  1≤i≤n,

(200) yi,kyj,kdj−aidi−aj≤0∀i,  1≤i,  j≤n,  k≠m+1,

(201)  yik=1,if  flight  i  is  assigned  to  gate  k,0<k≤m+1,0,otherwise,

(202)   ∀i,  1≤i≤n, ∀k,  1≤k≤m+1,

where constraint (199) ensures that every flight must be assigned to one and only one gate or assigned to the apron and constraint (200) requires that flights cannot overlap if they are assigned to the same gate.

Using the same case study by Ding et al. [6, 35], a greedy algorithm was designed to obtain an initial solution, which has been improved using simulated annealing (SA) and a hybrid of simulated annealing and tabu search (SA-TS).

#### 2.1.6. Multiple Objective AGAP Formulations

Hu and Di Paolo [36] mathematically formulated the multiobjective GAP (MOGAP) as a minimization problem and solved this problem using a new genetic algorithm with uniform crossover. The developed MOGAP model was presented as follows:

(203) $$\begin{array}{c} \underset{Q_{1},\ldots ,Q_{\mathrm{NG}}}{\text{Minimize}}\;J_{\text{MOGAP}} \end{array}$$

subject to

(204)  ∑g=1NGHg=NAC,

(205) EQgj=PQgj,  j=1max⁡⁡PQgj,EQgj−1,GQgj−1,  j>1,ffffffffGGGfffffj=1,…,Hg, g=1,…,NG,

(206)  Wi=Ei−Pi,   i=1,…,NAC,

(207)  JTPWD=∑g=1NG+1 ∑j=1Hg ∑i=1NAC+1MPQgj,iMPWDg,vi,

(208)  JTBTD=∑g=1NG+1 ∑j=1Xn ∑i=1NAC+1MPQgj,iMPTDg,vi,

(209)  JTPWT=∑g=1NACWi∑i=1NAC+1(MPi,j+MPj,i),

(210)  JMOGAP=αJTPWD+βJTBTD+1−α−βφJTPWT,

(211)  α+β≤1, 0≤α≤1, 0≤β≤1.

Wei and Liu [16] considered the AGAP as a fuzzy model and adopted a hybrid genetic algorithm to solve the developed model. The main objectives were minimizing passengers' total walking distance and gates idle times variance. They developed the following model:

(212)  minimize  Z1=∑i=1N ∑j=1N ∑k=1M+1 ∑l=1M+1fijTklyikyil,

(213)  minimize  Z2=∑i=1NSi2+∑k=1MSk2

subject to

(214)  ∑k=1Myik=1, ∀i∈N,

(215) dj−ai+αdi−aj+αyikyjk≤0,GG∀i,j=1,…,N, ∀k=1,…,M,

(216)  yik∈0,1, ∀i∈N,  ∀k∈K,

where objective function (212) reflects the total walking distance of passengers. *y*_*ik*_ is 0-1 variable; *y*_*ik*_ = 1 if flight *i* is assigned to gate *k*; otherwise it is 0; *f*_*ij*_ describes the number of passengers transferring from flight *i* to *j*, and *T*_*kl*_ is walking distance for passenger from gate *k* to *l*. Objective function (213) is used as a surrogate for the variance of idle times. The actual number of assignments is *N* and the number of nondummy idle times is *N* + *M*. Constraint (214) indicates that every flight must be assigned to one gate. Constraint (215) shows that flights that have overlap schedule cannot be assigned to the same gate, where *α* is the least safe time between continuous aircrafts assigned to the same gate. Constraint (216) denotes that *y*_*ik*_ is a binary variable.

In 2001, Yan and Huo [2] formulated the AGAP as a model with two objectives: minimizing (3) the walking distance, and (4) the waiting time for the passengers. The proposed mathematical model is binary integer linear programming:

(217a)  Minimize  Z1=∑i=1M ∑j=1N ∑k=BiLicij−xijk,

(217b)  Minimize  Z2=∑i=1MPi∑j=1N ∑k=BiLikxijk−Bi

subject to

(217c)  ∑j=1N ∑k=BiLixijk=1 ∀i

(217d)  ∑i∈Fj ∑k∈Hisxijk≤1 ∀j,  ∀S

(217e)  xijk=0  or  1, ∀i,  ∀j,  ∀k,

where objective (217a) represents the minimum total passenger walking distance. Objective (217b) represents the minimum total passenger waiting time. Constraint (217c) denotes that every flight must be assigned to one and only one gate. Constraint (217d) ensures that at most one aircraft is assigned to every gate in every time window.

Column generation approach, simplex method, and B&B algorithm were used to solve the proposed problem, which was a case study in Chiang Kai-Shek Airport, Taiwan. The problem consisted of 24 gates (of which two were temporary; eight out of 24 gates were only available for the wide type of aircrafts, whereas the rest were available for the other types) and 145 flights. The results showed that the obtained solution (7,300,660 s the best feasible solution found so far) was away from the optimal one by 0.077% (5595s).

Wipro Technologies [17] proposed a binary multiple objective integer quadratic programming model for the AGAP with a quadratic objective function. The proposed model was reformulated into a mixed binary integer linear programming model (linear objective functions and constraints). The proposed model has been solved using greedy heuristic, SA, and TS (MIP solvers based B&B cannot solve the proposed model within a reasonable time). The developed model was represented as follows.

*Generic Model.* Consider

(218)  Minimize∑i∈Nyi(m+1),

(219)  Minimize∑i,j∈N ∑k,l∈Kfijcclyikyjl

subject to

(220)   ∑k∈Kyik=1, ∀i∈N,

(221)   yik=0, ∀i∈N,  ∀k∈Ki,

(222)  yik>yjl, ∀i∈N,  ∀k∈K,  ∀j∈Ni,  ∀l∈Kk,

(223)  yik+yjl≤1, ∀i∈N,  ∀k∈K,  ∀j∈Ni,  ∀l∈Kk,

(224)   yik=∑j∈Nzijk, ∀i∈N,  ∀k∈K,

(225)  yik=∑i∈Nzijk, ∀j∈N,  ∀k∈K,

(226)  ti≥ai+α, ∀i∈N,

(227)  ti≤di−θi∗∑j∈Nfji, ∀i∈N,

(228)  ti+θi∗∑j∈Nfji≤tj+1−zijk∗M, ∀i,j∈N,

(229)  aj+1−zijk∗M≥di+β, ∀i,j∈N,  ∀k∈K,

(230)  yik,zijk∈0,1, ∀i,j∈N,  ∀k,l∈K,

(231)  ti≥0, ∀i∈N,

where objective function (218) aims at minimizing the number of flights that must be assigned to the apron, that is, those left ungated. Objective function (219) seeks to minimize the total connection times by passengers. Constraint (220) specifies that every flight must be assigned to one gate. Constraint (221) shows the equipment restriction on certain gates. Constraints (222) and (223) restrict the assignment of specific adjacent flights to adjacent gates. Constraints (224) and (225) indicate that every flight can have at most one flight immediately following and at most one flight immediately preceding, at the same gate. Constraints (226) and (227) stipulate that a gate must open for boarding on a flight during the time between its arrival and departure, and it also must allow sufficient time for handling the passenger/luggage boarding, which is assumed to be proportional to the number of passengers going on board. Constraint (228) ensures that each gate only serves one flight at any particular time (i.e., if flight *i* is assigned immediately before flight *j* to the same gate *k*, the gate must open for flight *i* earlier than for flight *j*). Constraint (229) further states the aircraft can only arrive at the gate when the previous flight has departed, while also including the buffer time between the flights. Constraints (230) and (231) specify the binary and nonnegative requirements for the decision variables.

*The Reformulated Model*. Minimizing (218) will remain the same:

(232) $$\begin{array}{c} \text{Minimize}\;\underset{i,j\in N}{\sum }\;\underset{k,l\in K}{\sum }f_{ij}c_{cl}x_{ijkl} \end{array}$$

subject to: Constraints… (220)–(231) from the generic model,

(233)  xijkl≤yik, ∀i,j∈N,  ∀k,l∈K,

(234)  xijkl≤yjl, ∀i,j∈N,  ∀k,l∈K,

(235) $$\begin{array}{c} y_{ik}+y_{jl}-1\leq x_{ijkl},\;\forall i,j\in N,\;\;\forall k,l\in K, \end{array}$$

(236)  xijkl∈0,1, ∀i,j∈N,  ∀k,l∈K,

where constraints (233), (234), and (235) specify that *x*_*ij* *kl*_ can be equal to one if and only if flight *i* is assigned to gate *k* and flight *j* is assigned to gate *l*. Constraint (236) expresses the binary requirement for the decision variable *x*_*ij* *kl*_.

Kaliszewski and Miroforidis [37] considered agap with the objective of assigning incoming flights to airport gates with some assumptions; those assumptions were as follows: gate assignment has no significant impact on passenger walking distance and no restrictions on the gates (all gates can take any type of airplanes) and neighboring gate operations can be carried out without any constraints. The model was stated as follows:

(237) minimize  f1x gggggggg=δmax⁡j⁡∑i=1mxj,iaj+1+2∑i=1mxj,iaj+2+⋯+α∑i=1mxj,iα

(238)  minimize  f2y=∑j=1nyj

subject to

(239)  ∑i=1m ∑t=ajΔxj,it≤1, for  j=1,…,n,

(240)  xj,it≤yiu for  u=t,  t+1,…,t+gj,

(241)  ∑j=1nxj,it≤1, t=1,…,Δ,  i=1,…,m,

(242)  ∑i=1m ∑t=ajΔxj,it≤1−yj, for  j=1,…,n.

#### 2.1.7. Stochastic Models

Yan and Tang [10] designed a framework for a stochastic AGAP (flight delays are stochastic). The framework included three main parts: the gate assignment model, a rule for the reassignments, and two adjustment methods for penalties. The performance of the developed framework has been evaluated using simulation-based evaluation method.

The formulation of the stochastic gate assignment model (the objective was to minimize the total waiting time of the passengers) was addressed as follows:

(243) Minimize  ZP=∑k∈K ∑ij∈Akcijkxijk +∑s∈Ωps∑k∈K ∑ij∈QAkuijs,kxijk+w∑s∈Ωpshs

subject to

(244)  ∑j∈Nkxijk−∑r∈Nkxrik=0 ∀i∈Nk,  ∀k∈K,

(245)  ∑k∈K ∑ij∈Ftxijk=1 ∀t∈AF,

(246)  0≤xijk≤gk ∀i,j∈CAk,  ∀k∈K,

(247)  ∑k∈K ∑ij∈QAkuijs,kxijk−hs≤∑s∈Ωps∑k∈K ∑ij∈QAkuijs,kxijk ∀s∈Ω,

(248)  hs≥0 ∀s∈Ω,

(249)  xijk∈Z+ ∀i,j∈CAk,  ∀k∈K,

(250)  xijk=0,1 ∀i,j∈Ak−CAk,  ∀k∈K,

where function (243) denotes the minimization of the total passenger waiting time, the expected penalty value for all *n* scenarios, and the expected semideviation risk measure (SRM) for all *n* scenarios multiplied by the weighting vector *w*. Constraint (244) is the flow conservation constraint at every node in each network. Constraint (245) denotes that every flight is assigned to only one gate and one time window. Constraint (246) ensures that the number of gates used in each network does not exceed its available number of gates. Constraints (247) and (248) are used to calculate the SRM. Constraint (249) ensures that the cycle arc flows are integers. Constraint (250) indicates that, except for the cycle arcs, all other arc flows are either zero or one.

The value of the performance measure (the objective, minimizing the total waiting time of the passengers) for each scenario in the real time stage was calculated as follows:

(251)  zrs=wts+its,

(252) ZR=AWT+AIT+w×SRMR=∑s=Ωpswts+∑s=Ωpsits +w∑s=Ωpsmax⁡⁡0,zrs−∑s=Ωpszrs=∑s=Ωpszrs+w∑s=Ωpsmax⁡⁡0,zrs−∑s=Ωpszrs.

For each iteration, the penalties were calculated, using the developed two adjustment methods for penalties, as follows.

*Method 1.* Consider

(253) $$\begin{array}{c} {\left(u_{ij}^{s,k}\right)}^{m+1}={\left(u_{ij}^{s,k}\right)}^{m}+{\left({dw}_{ij}^{s,k}+{di}_{ij}^{s,k}\right)}^{m}. \end{array}$$

*Method 2.* Consider

(254) dvijs,km=1,if  dwijs,k>0  or  diijs,k>00,if  dwijs,k=0,  diijs,k=0∀i,j∈QAk, ∀k∈K, ∀s∈Ω,

(255)   bm=max⁡0,−∑dvijs,kmdijs,km−1dijs,km−12,

(256) dijs,km=dvijs,km+bmdijs,km−1 ∀i,j∈QAk,hhhhhhhjhhhhhhhhhhhhh∀k∈K,∀s∈Ω

(257)   tm=λZRm−ZPmdvijs,k2, 0<λ≤2,

(258) uijs,km1=uijs,km +tmdijs,km ∀i,j∈QAk,  ∀k∈K,  ∀s∈Ω.

The data was taken from the Chiang Kai-Shek (CKS) airport (172 flights, 2 gate types, and 14 aircraft types); the distributions for the flight delays were obtained from the actual data taken from the CKS airport. The obtained results were 197 minutes which was the longest solution time of the framework, which was efficient in the planning stage, but after 40 scenarios, the solution times increased significantly but the solution results were more stable.

Genç et al. [38] developed a stochastic model for AGAP with the objective of minimizing the gate duration, gate duration defined as the total time of the allocated gates (for all flights in a day):

(259) $$\begin{array}{c} F_{\text{fitness}}=\overset{Ng}{\underset{k=1}{\sum }}\;\overset{N_{t}}{\underset{l=1}{\sum }}\text{any}\left(M_{c}\left(k,l\right)\right) \end{array}$$

subject to

(260) $$\begin{array}{c} \text{any}\left(M_{c}\left(k,l\right)\right)=\left\{\begin{array}{cc} 1, & \text{if}\;M_{c}\left(k,l\right)\neq 0\\ 0, & \text{otherwise}. \end{array}\right. \end{array}$$

Şeker and Noyan [9] also developed a stochastic model considering the minimization of the number of conflicts and the expected variance of the idle times as a performance measure; the proposed performance measure was a part of the mixed integer programming model presented in Section 2.1.3:

(261) $$\begin{array}{c} \text{Minimize}\;\underset{s\in S}{\sum }V_{s}p_{s}+\Lambda \underset{i\in N^{b}}{\sum }\;\underset{k\in M}{\sum }\;\underset{s\in S}{\sum }\left(c_{i,k,s}^{p}+c_{i,k,s}^{f}\right)p_{s} \end{array}$$

subject to

(262)  ∑k∈Mxi,k=1, i∈N,

(263)  x0,k=1, k∈M,

(264)  xn+k,k=1, k∈M,

(265)  ∑j∈Li,sxj,k+xi,k≤1, i∈Nb,  k∈M,  s=0,

(266) ci,k,sp≥∑j∈Li,spxj,k+xi,k−1n+m,ffffggffi∈Nb, k∈M, s=S,

(267) ci,k,sf≥∑j∈Li,sfxj,k+xi,k−1n+m,hhffffhhi∈Nb, k∈M, s=S,

(268) Ai,s≤2−xi,k−xj,kZ+aj,s,hhhhhhhhi∈Nb∖0, j∈Li,s′, k∈M, s=S,

(269) Ai,s≤2−xi,k−xj,kZ+aj,s,i∈Nb∖0, j∈Li,sp, k∈M, s=S,

(270) Ai,s≤2−xi,k−xj,kZ+aj,s,i∈Nb∖0, j∈Li,sf, k∈M, s=S,

(271)  ∑i∈Nb∖0Ai,s=∑i∈Nai,s+a0,sm, s=S,

(272)  Ii,s=Ai,s−di,s+si,sp+si,sf, i∈Nb∖0,  s=S,

(273) Ii,s≤2−xi,k−xj,kZ,i∈Nb∖0, j∈Li,sp, k∈M, s=S,

(274) Ii,s≤2−xi,k−xj,kZ, i∈Nb∖0,gggghhhhggggj∈Li,sf, k∈M,  s=S,

(275)  si,sp≤Z∑k∈Mci,k,sp, i∈Nb,  s=S,

(276)  si,sf≤Z∑k∈Mci,k,sf, i∈Nb,  s=S,

(277)  xi,k∈0,1,   i∈Nb,  k∈M,

(278)  All  remaining  variables≥0.

### 2.2. AGAP Related Problems

In some of the publications on the GAP the researchers have formulated the AGAP as well-known related problems such as quadratic assignment problem (QAP), clique partitioning problem (CPP), and scheduling problem or even as a network representation. However, some of the researchers formulated the AGAP as a robust optimization model. In this section, we will present the work that has been done on the GAP as a well-known related problem.

#### 2.2.1. Quadratic Assignment Problem (QAP)

Drexl and Nikulin [3] modeled the multicriteria airport gate assignment as quadratic assignment problem (QAP) and solved the problem using Pareto simulated annealing. The performance measures were as follows: minimizing connection times or total passenger walking distances, maximizing the preferences of total gate assignment, and minimizing the number of ungated flights:

(279a) min⁡z1=∑i=1nπi,m+1,

(279b) min⁡⁡z2=∑i=1n ∑j=1n ∑k=1m+1 ∑l=1m+1fi,jwk,lπi,kπj,l+∑i=1n ∑k=1m+1f0,iw0,kπi,k+∑i=1n ∑k=1m+1fi,0wk,0πi,k,

(279c)  min⁡z3=∑i=1n ∑k=1m+1viui,kπi,k

subject to

(280)  ∑k=1m+1πi,k=1, 1≤i≤n,

(281)  πi,kπj,kdj−aidi−aj≤0, 1≤i,  j≤n,  k≠m+1,

(282)  πi,k∈0,1, 1≤i≤n,  1≤k≤m+1,

where objective (279a) addresses the number of flights that are not assigned to any terminal gate (i.e., to the apron). Objective (279b) represents the total passenger walking distance. It consists of three terms: the walking distance of transfer passengers, originating departure passengers, and disembarking arrival passengers. Objective (279c) represents the total value for flight gate assignment preference. Constraint (280) ensures that every flight must be assigned to exactly one gate including the apron. Constraint (281) prohibits schedule overlapping of two flights if they are assigned to the same terminal gate. Constraint (282) defines the variables to be Boolean.

Haghani and Chen [13] modeled the AGAP as QAP with minimizing the total passenger walking distances (transfer passengers and local passenger) as a performance measure. The QAP model was expressed as follows:

(283) Minimize  ∑i ∑i′ ∑j ∑j′Pii′Djj′XijtXi′j′t′ fffffffffff+∑i ∑i′Pi0Dj0+P0iD0jXijt,

(284)   Pii′Djj′⟵Pi0Dj0+P0iD0j ∀i,j,

(285)  Minimize  ∑i ∑i′ ∑j ∑j′Pii′Djj′XijtXi′j′t′.

According to the simplicity of solving linear models, the previous model was transformed into a linear model as follows:

(286)  Yiji′j′=XijtXi′j′t′,

(287)  Yiji′j′=1,if  flight  i  is  assigned  to  gate  jand  flight  i′  is  assigned  to  gate  j′,0,otherwise,

(288)  Minimize  ∑i ∑i′ ∑j ∑j′Pii′Djj′Yiji′j′

subject to

(289)  ∑jXijt=1 ∀i,  tia≤t≤tid,

(290)  ∑iXijt≤1 ∀j,t,

(291)  Xijt≤Xijt+1 ∀t,  tia≤t≤tid−1,

(292)  Xijtia+Xijti′a−2Yiji′j′a≥0 ∀i<i′,j,j′,

(293)  ∑j ∑j′Yiji′j′=1 ∀i≠i′.

The results and conclusions were as follows: the proposed approach was efficient which provided results close to the optimal solution according to the percent of improvement from the starting solution (initial solution), in the case of 10 flights and 10 gates, 20 flights and 5 gates, and 30 flights and 7 gates.

#### 2.2.2. Scheduling Problems

In 2010, Li [39] formulated the GAP as a parallel machines scheduling problem and used the dynamic scheduling and the direct graph model to solve the proposed model; B&B was used in solving the small size problems while the large size problems have been solved using dynamic scheduling.

#### 2.2.3. Clique Partitioning Problem (CPP)

Dorndorf et al. [8] developed an optimization model for the GAP and transformed that model into a CPP model; the two models are written below. A heuristic approach that was developed by Dorndorf and Pesch (1994), based on the ejection chain algorithm, has been used to solve the transformed model (CPP model).

*The Optimization Model for the AGAP.* Consider

(294) $$\begin{array}{c} \underset{\left\{f\;|\;f:N\to M\right\}}{\text{Minimize}}\;\;\alpha_{1}z_{1}\left(f\right)+\alpha_{2}z_{2}\left(f\right)+\alpha_{3}z_{3}\left(f\right) \end{array}$$

subject to

(295)  fi∈Mi ∀i∈N,fi≠fj ∀tij<0,fi≠n+m, ∀i,j∈N, fi≠k∨fi≠l ∀i,k,j,l∈S,

(296)  z1=−∑i=1npifi∗, z2=i∈N ∣ U(i)≠0∧f(i)≠f(Ui), z3=∑(i,j) ∣ i<j,fi=f(j)≠n+mmax⁡⁡tmax⁡−tij,0.

*The CPP Transformation of the Problem.* Consider

(297) $$\begin{array}{c} \text{maximize}\underset{1\leq i<j\leq a}{\sum }w_{ij}x_{ij} \end{array}$$

subject to

(298) xij+xjk−xik≤1 for  1≤i<j<k≤a, xij−xjk+xik≤1 for  1≤i<j<k≤a, −xij+xjk+xik≤1 for  1≤i<j<k≤a,    xij∈0,1 for  1≤i<j≤a,(299) V≔1,2,…,n+m−1,(300)wij≔−∞if  tij<0α2if  tij≥0  ∧Ui=j∨Uj=i−α3·max⁡⁡tmax⁡−tij,0if  tij≥0∧Ui≠j  ∧  Uj≠i  ∀i,j<n,(301)wij≔−∞if  j∉Miα1·pij∗if  j∈Mi  mmggggm∀i≤n, j>n(302) wij≔−∞    ∀i,j>n.

#### 2.2.4. Network Representation

Maharjan and Matis [40] formulated the GAP as a binary integer multicommodity network flow model with minimizing the passengers comfort and aircraft fuel burn as a performance measure. For passengers comfort and with arguments of distance and time for connection a penalty function in three dimensions was specified. For large size problem and based on a methodology of zoning a decomposition approach was provided and compared with the assignments made by the airline, and the results showed that the developed methodology was shown to be computationally efficient.

*The Mathematical Formulation.* Consider

*X* _*Si*_ ^*k*
^
: binary variable representing initial assignment of gate *k* ∈ **K** to aircraft *i* ∈ **F**;
*X* _*ji*_ ^*k*
^
: binary variable representing assignment of gate *k* ∈ **K** to aircraft *j* ∈ **D** followed by *i* ∈ **A**;
*X* _*jT*_ ^*k*
^
: binary variable representing last assignment of gate *k* ∈ **K** to aircraft *j* ∈ **D**;
*X* _*ST*_ ^*k*
^
: binary variable representing no assignment of gate *k* ∈ **K** to any aircraft:

(303) Minimize  Z=∑i∈A ∑k∈KCk+2fifcfsdk1+dk2Xs,ik+∑j∈DXj,ik +∑(i∈F,i=j,i′=j′,i≠i′,j≠j′) ∑(i′∈F,i=j,i′=j′,i≠i′,j≠j′) ∑k∈K  ∑k′∈KNii′Ckk′ii′Xi,jkXi′,j′k′

subject to

(304)  ∑iXs,ik+XS,Tk=1 ∀k∈K,  i∈F,

(305) Xs,ik+∑iln⁡ijXj,ik=Xm,nk ∀i,m∈A, j,n∈D, fffffffffffffffffffffffffffffffffffffff i≠j, m=n,

(306) ∑iln⁡ijXj,ik+Xj,Tk=Xm,nk ∀i,m∈A, j,n∈D, dddddddddddddddddddd i≠j, m=n,

(307)  ∑iXjTk+∑iXSTk=1 i∈F,  k∈K,

(308)  ∑iXijk=1 ∀i∈A,  j∈D,  i=j,

(309)  Xs,ik,Xj,ik,Xm,nk,XS,Tk,XjTk=0,1,

(310)  ln⁡ij=1,if  Ai−Dj≥p    ∀i≠j0,otherwise,

where (303) represents the expected taxi in and out fuel burn cost of assigning a plane to a particular gate based on the expected runway distance corresponding to arrival and departure cities for the flight. Equation (304) is referred to as a flow-in constraint because it deals with the gate flow from the source node to the arrival flight node. Equation (305) is referred to as conservation of flow at the arrival node. Equation (306) is conservation of flow at the departure node. Equation (307) is the flow-out constraint that forces all the flow to leave the departure node to the terminal node. Equation (308) is referred to as a unit flow serving arc constraint as it allows only one unit gate *k* ∈ *K* to flow through serving arc. Equation (309) is the binary constraints.

The above model was with a quadratic objective function and a linearization for that objective was made as follows:

(311) $$\begin{array}{c} \text{Minimize}\;Z_{2}=\underset{\left(i\in F,i=j,i^{\prime }=j^{\prime },i\neq i^{\prime },j\neq j^{\prime }\right)}{\sum }\;\underset{\left(i^{\prime }\in F,i=j,i^{\prime }=j^{\prime },i\neq i^{\prime },j\neq j^{\prime }\right)}{\sum }\;\underset{k\in K}{\sum }\;\underset{k^{\prime }\in K}{\sum }N_{ii^{\prime }}C_{kk^{\prime }}^{ii^{\prime }}X_{i,j}^{k}X_{i^{\prime },j^{\prime }}^{k^{\prime }}. \end{array}$$

The linearization has been made by replacing the quadratic term (*X*_*i*,*j*_^*k*^ *X*_*i*′,*j*′_^*k*′^) by a new variable *Y*_*ii*′_^*kk*′^ defined as follows:

(312) Yii′kk′=1,if  Xijk=1,  Xi′j′k′=1  ∀i=j,gggi′=j′,i≠i′,j≠j′,  k≠k′Xi′j′k′0,otherwise

subject to

(313)  Yii′kk′−Xijk≤0 ∀i=j,  i′=j′,  i≠j′,  k≠k′,

(314)  Yii′kk′−Xi′j′k′≤0 ∀i=j,  i′=j′,  i≠j′,  k≠k′,

(315)  Xijk+Xi′j′k′−Yii′kk′≤1 ∀i=j,  i′=j′,  i≠j′,  k≠k′,

(316)  Xijk+Xi′j′k′−Yii′kk′≥0 ∀i=j,  i′=j′,  i≠j′,  k≠k′,

where inequalities (313) and (314) indicate that variable *Y*_*ii*′_^*kk*′^ is equal to 1 if and only if binary variables *X*_*ij*_^*k*^ and *X*_*i*′*j*′_^*k*′^ are equal to 1. Equation (315) specifies that *Y*_*ii*′_^*kk*′^ cannot be greater than 1, and (316) further specifies that *Y*_*ii*′_^*kk*′^cannot be less than zero. Due to the binary nature of *X*_*ij*_^*k*^ and *X*_*i*′*j*′_^*k*′^ with the above constraints, *Y*_*ii*′_^*kk*′^ is forced to be a binary variable.

According to the passengers comfort, a cost function (*C*_*ii*′_^*kk*′^) was represented as follows:

(317) $$\begin{array}{c} C_{ii^{\prime }}^{kk^{\prime }}=\left\{\begin{array}{cc} s\sqrt{d_{kk^{\prime }}}\;{\left(2-\Delta t_{ii^{\prime }}\right)}^{2}, & \forall 0<\Delta t_{ii^{\prime }}\leq t_{\max}\\ 0, & \text{otherwise}, \end{array}\right. \end{array}$$

where (317) is a surface plot of the cost function.

*Mathematical Formulation Using the Methodology of Zoning.* Consider

(318) Minimize  Z=∑i∈F ∑k∈Kz2fifcfsdk1+dk2Xs,ik+∑j∈DXj,ik +∑(i∈F,i=j,i′=j′,i≠i′,j≠j′) ∑(i′∈F,i=j,i′=j′,i≠i′,j≠j′) ∑k∈Kz ∑k′∈KNii′Ckk′ii′XijkXi′j′k′

subject to the following.

The constraints of (304) and (308)   are modified and replaced with

(319)  ∑iXsik+XSTk=Kz ∀k∈Kz,  i∈F,

(320)  ∑jXjTk+∑kXSTk=Kz ∀i∈F,    k∈Kz.

In order to solve the developed model a code for the developed model was written using an AMPL/CPLEX 11.2 package, and as mentioned before the results showed that the developed methodology was shown to be computationally efficient.

### 2.3. Robust Optimization

Diepen et al. [41] formulated a completely new integer linear programming formulation for the GAP with a robust objective function that is based on the so-called gate plans. The objective was to maximize the robustness of a solution, which can be expressed as an allocation of a maximum possible idle time between each pair of consecutive flights to guarantee that each flight can afford to land with some slight earliness or tardiness without the need for re-planning the schedule:

(321) $$\begin{array}{c} x_{j}=\left\{\begin{array}{cc} 1, & \text{if}\;\text{gate}\;\text{plan}\;j\;\text{is}\;\text{selected}\\ 0, & \text{otherwise}, \end{array}\right.\\ \text{Minimize}\;\overset{N}{\underset{i=1}{\sum }}c_{i}x_{i} \end{array}$$

subject to

(322)  ∑i=1Ngvixi=1 for  v=1,…,V,

(323)  ∑i=1Neiaxi=Sa for  a=1,…,A,

(324)  xj∈0,1 for  j=1,…,n,

where

(325)  gvi=1,if  flight  v  is  in  gate  plan  i,0,otherwise, eia=1,if  flight  i  is  of  type  a,0,otherwise.

In addition, to add the preferences to the ILP model the following constraints have been added to the model:

(326) $$\begin{array}{c} \overset{N}{\underset{i=1}{\sum }}\;\overset{V}{\underset{v=1}{\sum }}\;\overset{A}{\underset{a=1}{\sum }}p_{vak}e_{ia}g_{vi}x_{i}\geq P_{k}\;\text{for}\;k=1,\ldots ,K, \end{array}$$

where

(i)
$p_{vak}=\left\{\begin{array}{cc} 1, & \text{if}\;\text{flight}\;v\;\text{has}\;\text{preference}\;\text{for}\;\text{gate}\;\text{type}\;a\;\text{in}\;\text{preference}\;k\\ 0, & \text{otherwise}; \end{array}\right.$

(ii) *P*_*k*_ denotes the minimum number of flights that have to be assigned to a given gate type;
(iii) according to preference *k*;
(iv) *K* denotes the total number of preferences:
(327) $$\begin{array}{c} \overset{N}{\underset{i=1}{\sum }}g_{vi}x_{i}+{UAF}_{v}=1\;\text{for}\;v=1,\ldots ,V, \end{array}$$

where *UAF*_*v*_ ≥ 0 for *v* = 1,…, *v*. and *UAF*_*v*_ is a penalty variable:

(328) ∑i=1Ngvi+gvA,ixi+UAFvA=1,∑i=1Ngvi+gvA,ixi+UAFvA=1,pihr =1,if  flight  v  has  preference  on⁡  gate  type  a  in  preference  k,0.5,if  the  split  version  of  flight  v  has  preferenceon⁡  gate  type  a  in  preference  k,0,otherwise.

The integrality of the developed model has been relaxed for the integrality and the resulting relaxed LP was exploited to obtain solutions of ILP by using column generation (CG).  Table 1 summarizes all the above mathematical formulations used recently for the AGAP.

## 3. Resolution Methods

As mentioned before in Section 2, most of the solution techniques presented in Sections 3.2 and 3.3 have been used concurrently with complex mathematical formulations that led to very high computing time. Section 3.1 addressed the exact solution techniques and the optimization programming language used to solve the proposed models to their optimality. Sections 3.1, 3.2, and 3.3 include the research work that has been done on the exact, heuristic, and metaheuristic approaches for solving the AGAP.

### 3.1. Exact Algorithms

Exact algorithms are those that yield an optimal solution. According to the literature, different exact solution techniques have been used to solve the GAP. As an example branch and bound was used as well as column generation and other methods, and in some research, the authors used some optimization programming languages like CPLEX and AMPL. In this section, only the research work that has been done on the exact solution techniques for solving the AGAP is presented.

Li [5, 32] solved the proposed hybrid mathematical model using CPLEX software. Mangoubi and Mathaisel [11] relaxed the integrality of the developed ILP model and solved the relaxed ILP model using CG; an optimal solution has been obtained for minimizing the total walking distance. Bihr [12] proposed a primal-dual simplex algorithm to find the solution and found the optimal solution. Yan and Huo [2] used simplex algorithm with column generation and weighting method to solve the provided model. Bolat [30, 34], Li [39], and Yan and Huo [2] used branch and bound algorithm to solve the models they have developed. Reference [14] used branch and bound algorithm and compared the result with tabu search algorithm.

### 3.2. Heuristic Algorithms

Basically the GAP is a QAP and it is an NP-hard problem as shown in Obata [21]. Since the AGAP is NP-hard, researchers have suggested various heuristic and metaheuristics approaches for solving the GAP. This section is for the heuristic algorithms; with heuristic algorithms, theoretically there is a chance to find an optimal solution. That chance can be remote because heuristics often reach a local optimal solution and get stuck at that point, so it was necessary to have modern heuristics called metaheuristic. This approach will be presented in the following part in this section; the research work that has been done on the heuristic approaches for solving the AGAP is presented.

Yan and Tang [10] developed a framework designed to deal with the GAP which has stochastic flight delays; the developed framework was with a heuristic approach embedded in it. Genç [42] used several heuristics, which are the “Ground Time Maximization Heuristic,” “Idle Time Minimization algorithm,” and “Prime Time Heuristic,” to solve the GAP with minimizing the idle gate time (or maximizing the number of assigned flights) as a performance measure. Ding et al. [6, 35] designed a greedy algorithm for solving the GAP with the objective of minimizing the number of ungated flights. Lim et al. [24] used several solution approaches, which are the “Insert Move Algorithm,” the “Interval Exchange Move Algorithm,” and a “Greedy Algorithm,” to solve the developed model for the GAP. Yan et al. [29] proposed a simulation framework and developed an optimization model (Section 2.1.2) and then solved the model using two greedy heuristics: the first was related to the number of passengers and the second was related to the arrival time of the flight at the gate. The distance result showed that the optimization model outperformed the heuristic but the heuristic simulation time outperformed the optimization model. Diepen et al. [25] solved the resulting LP-relaxation and the original ILP model using column generation.

Thengvall et al. [43] represented a heuristic approach for the problem of schedules recovery in airports during hub closures; the proposed approach was a bundle algorithm approach. Dorndorf et al. [8] used a heuristic approach that was developed by Dorndorf and Pesch (1994), which was based on the ejection chain algorithm, to solve the transformed model (CPP model). Mangoubi and Mathaisel [11] also used heuristic approach to solve the GAP with the objective of minimizing walking distance for the passengers and the obtained solutions were compared with the optimal solution using LP to obtain the deviation in the results. Haghani and Chen [13] used a heuristic approach to solve the GAP with the objective of minimizing walking distance for the passengers. Vanderstraeten and Bergeron [28] developed a direct assignment of flights to gates algorithm, named ADAP; the developed algorithm was an implicit enumeration which has been faster by carefully applying some variables selection criteria, which are the concept of “main chain,” the assigned weights to variables, and the single assignment constraints. Bolat [30] used branch and trim heuristic to solve the GAP with the objective of minimizing the slack times range. Bolat [34] used the HBB and SPH heuristics to solve the models that he has developed for the GAP. The first approach (HBB) is a B&B approach that has been utilized by some restrictions on the number of the nodes that has to be branched in a search tree while the second approach (SPH) developed a heuristic that after *N* iterations builds only one solution. The used heuristics assigned flights one at a time by considering all available gates and for determining the most permissible gate a priority function was utilized.

### 3.3. Metaheuristic Algorithms

As mentioned before in Section 3.2, heuristics often get stuck in a local optimal solution, but metaheuristics or “modern heuristics” introduce systematic rules to deal with this problem. The systematic rules avoid local optima or give the ability of moving out of local optima. The common characteristic of these metaheuristics is the use of some mechanisms to avoid local optima. Metaheuristics succeed in leaving the local optimum by temporarily accepting moves that cause worsening of the objective function value. In this section, the research work that has been done on the metaheuristic approaches for solving the AGAP is presented.

Gu and Chung [44] introduced a genetic algorithm model to solve the AGAP. The developed model has been implemented in a high-level programming language; the effectiveness of the developed model has been validated by testing different scenarios and the results showed that the performance of the developed model was efficient. Şeker and Noyan [9] developed stochastic programming models; the developed model has been formulated as a mixed integer programming but in large scale. The developed models were solved using tabu search (TS) algorithm, and the obtained results were with high quality.

Cheng et al. [23] studied the performance of several metaheuristics in solving the GAP. The metaheuristics were genetic algorithm (GA), tabu search (TS), simulated annealing (SA), and a hybrid of SA and TS. Tabu search (TS) outperforms SA and GA but the hybrid approach outperforms TS in terms of solution quality. Ding et al. [6, 35] used a tabu search algorithm to solve the GAP; the starting (initial) solution was obtained using a designed greedy algorithm. Xu and Bailey [14] developed a tabu search algorithm to solve the GAP and compared the result of the developed algorithm with a branch and bound algorithm. The results showed that the two approaches provide optimal solutions for the studied problems but TS obtained was better in the CPU time.

Zheng et al. [33] developed a model for solving the GAP (see Section 2) and used a TS algorithm to obtain solutions for the developed model. Ding et al. [7] used a simulated annealing and a hybrid of SA and TS to solve the GAP model that they have developed; the starting (initial) solution was obtained using a designed greedy algorithm. Lim et al. [24] proposed TS and memetic algorithms to solve the GAP. Drexl and Nikulin [3] solved the multicriteria airport gate assignment using Pareto simulated annealing. Hu and Di Paolo [36] solved the multiobjective gate assignment problem (MOGAP) using a new genetic algorithm with uniform crossover. Bolat [31] used genetic algorithm (GA) to minimize the variance or the range of gate idle time. Wei and Liu [16] modified a hybrid genetic algorithm to solve the fuzzy AGAP model.  Table 2 summarizes all the above solution techniques used recently for the AGAP.

Recently, Bouras et al. [45] approached the AGAP as a parallel machine-scheduling problem with some priority and eligibility. They solved the problem with the aim of minimizing the following objectives: total cost, total tardiness, and maximum tardiness. They developed three heuristics and used three metaheuristics (simulated annealing, tabu search, and genetic algorithms). The evaluation was conducted over 238 generated instances but only 50 instances were presented in the report. The results showed that simulated annealing was the most efficient metaheuristic to solve the problem.

## 4. Conclusion and Research Trends

In this survey, we have presented the very recent publications about the airport gate assignment problem. The collected literature has the aim of identifying the contributions and the trends in the research using exact or approximate methods.

An abundant literature is listed to describe mathematical formulation on AGAP or other related problems. They have been grouped in such a way that the user is guided to identify each problem specification. For single objective, integer/binary models are described along with mixed integer ones. Nonlinear formulations are also described for mixed/integer models. Rare are the authors who really came out with exact solutions using existing commercial optimization software or their own exact methods (branch and bound…). Heuristics were suggested to build feasible solutions and improve the latter solutions using metaheuristics.

For multiobjective optimization, several models have been formulated as nonlinear objectives with little success in solving such problems with exact methods in a reasonable time.

Related problems to AGAP have also been introduced in the survey. Some cases of AGAP have been formulated as some well-known combinatorial optimization problems such as QAP.

**Figure 1:**
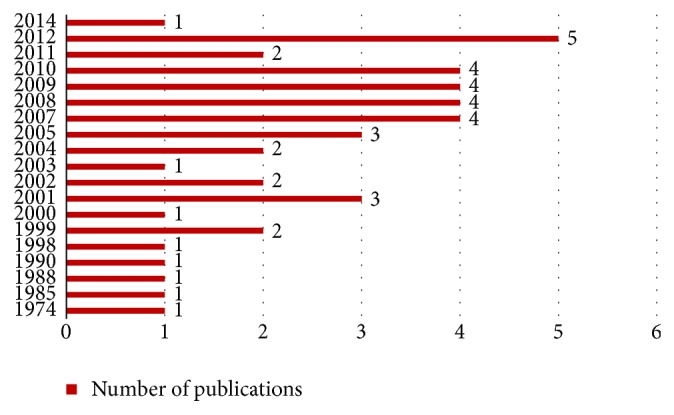
Number of publications per year.

**Figure 2:**
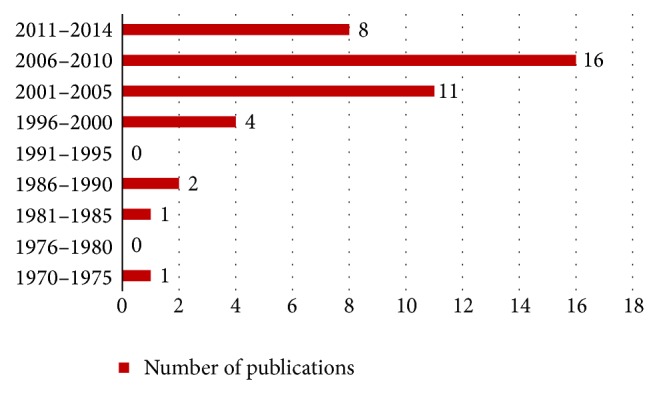
Number of publications per 5 years.

Since 2005 (Table 3, Figures 1-2), most of the people started to consider using heuristics/metaheuristics as tools to solve AGAP since the problem is NP-hard, and the issue of time solving was still unresolved by the existing tools.

In practice, major airlines may have more than 1000 daily flights to handle at more than 50 gates, which results in billions of binary variables in formulation. B&B based MIP solvers (i.e., CPLEX) will not be able to handle such huge size problems within a reasonable time bound.

**Figure 3:**
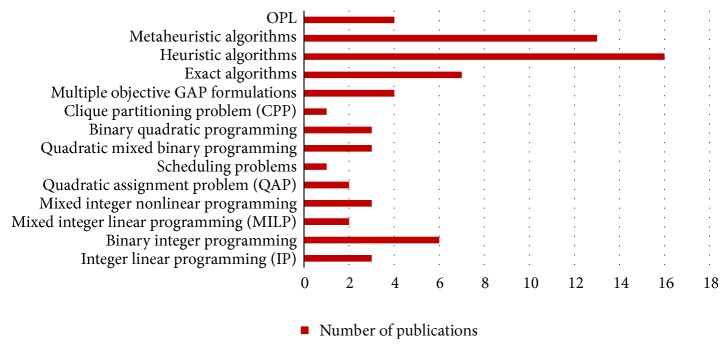
Number of publications per research area.

A growing interest in metaheuristics (Table 4, Figure 3) has been observed in the recent papers on AGAP. TS, SA, and GA are the most used improvement methods. Some hybridized methods combining these methods have also been suggested.

It is quite understandable that researches move towards the use of such methods. Airport managers usually face changes on their plans and need to change their plans due to uncertainties.

With a lack of studies on robust methods or stochastic methods, where only few papers appeared on these subjects, researchers have at their disposal a battery of methods such as evolutionary methods, parallel metaheuristics, and self-tuning metaheuristics to apply on this interesting problem.

With the actual trend of heuristics use, it would be interesting to work on a combination of these algorithms

In the framework of a hyperheuristic approach, many hard combinatorial optimization problems have already been tackled using heuristics (Burke et al. [46]), which motivates our recommendation of this approach. Strengthening weaknesses is the essence of hyperheuristics since they smartly work with search spaces of heuristics. The idea is, during a process of exploring new solutions, to choose the adequate metaheuristic where the currently used one is failing to improve or to generate new heuristics by using the components of existing ones (Soubeiga [47]; Burke et al. [48]).

There is an alternative to these approximate methods, which could be explored: the use of efficient exact methods (branch and bound…) with the introduction of tight lower bounds. In our review, we found that only Tang et al. [27] developed a lower bound and used a classical branch and bound algorithm, while other authors combined special heuristics with their method (Bolat [30, 34], Li [39], and Yan and Huo [2]).

We have also noticed the absence of a data set for AGAP. It could be interesting to have a set of instances of different sizes that can be shared by researchers, with benchmarks (optimal and best-known values) and CPU times to help comparing methods as it is the case for known problems: quadratic assignment problem and travelling salesman problem.

## Figures and Tables

**Table 1:** Formulations of AGAP and related problems.

Formulation	References	Criterion (comments)	Problem type
Integer linear programming (IP)	Lim et al. [24]	(i) Minimizing the sum of the delay penalties (ii) Minimizing the total walking distance	Theoretical
	Diepen et al. [25]	(i) Minimizing the deviation of arrival and departure time (ii) Minimizing replanning the schedule	Real case (Amsterdam Airport Schiphol)
	Diepen et al. [26]	Minimizing the deviations from the expected arrival and departure times	Real case (Amsterdam Airport Schiphol)

Binary integer programming	Mangoubi and Mathaisel [11]; Yan et al. [29]	Minimizing passenger walking distances	Real case (Toronto International Airport); Real case (Chiang Kai-Shek Airport)
	Vanderstraeten and Bergeron [28]	Minimizing the number off-gate event	Theoretical
	Bihr [12]	Minimizing of the total passenger distance	Theoretical
	Tang et al. [27]	Developing a gate reassignment framework and a systematic computerized tool	Real case (Taiwan International Airport)
	Prem Kumar and Bierlaire [18]	(i) Maximizing the gate rest time between two turns (ii) Minimizing the cost of towing an aircraft with a long turn (iii) Minimizing overall costs that include penalization for not assigning preferred gates to certain turns	Theoretical

Mixed integer linear programming (MILP)	Bolat [30]	Minimizing the range of slack times	Real case (King Khaled International Airport)
	Bolat [31]	Minimizing the variance or the range of gate idle time	Real case (King Khaled International Airport)

Mixed integer nonlinear programming	Li [5, 32]	Minimizing the number of gate conflicts of any two adjacent aircrafts assigned to the same gate	Real case (Continental Airlines, Houston Gorge Bush Intercontinental Airport)
	Bolat [31]	Minimizing the variance or the range of gate idle time	Real case (King Khaled International Airport)

Multiple objective GAP formulations	Hu and Di Paolo [36]	Minimize passenger walking distance, baggage transport distance, and aircraft waiting time on the apron	Theoretical
	Wei and Liu [16]	(i) Minimizing the total walking distance for passengers (ii) Minimizing the variance of gates idle times	Theoretical
	B.A.C.o.E.B. Team and A.I.C.o.E. Team [17]	(i) Minimizing walking distance (ii) Maximizing the number of gated flights (iii) Minimizing flight delays	Theoretical
	Yan and Huo [2]	(i) Minimizing passenger walking distances (ii) Minimizing the passenger waiting time	Real case (Chiang Kai-Shek Airport)
	Kaliszewski and Miroforidis [37]	Finding gate assignment efficiency which represents rational compromises between waiting time for gate and apron operations	Theoretical

Stochastic model	Yan and Tang [10]	Minimizing the total passenger waiting time	Real case (Taiwan International Airport)
	Genç et al. [38]	Maximizing gate duration, which is total time of the gates allocated	Theoretical and real case (Ataturk Airport of Istanbul, Turkey)
	Şeker and Noyan [9]	Minimizing the expected variance of the idle time	Theoretical

Quadratic assignment problem (QAP)	Drexl and Nikulin [3]	(i) Minimizing the number of ungated flights (ii) Minimizing the total passenger walking distances or connection times (iii) Maximizing the total gate assignment preferences	Theoretical
	Haghani and Chen [13]	Minimizing the total passenger walking distances	Theoretical

Scheduling problems	Li [39]	(i) Maximizing the sum of the all products of the flight eigenvalue (ii) Maximizing the gate eigenvalue that the flight assigned	Theoretical

Quadratic mixed binary programming	Bolat [34]	Minimizing the variance of idle times	Real case (King Khaled International Airport)
	Zheng et al. [33]	Minimizing the overall variance of slack time	Real case (Beijing International Airport, China)
	Xu and Bailey [14]	Minimizing the passenger connection time	Theoretical

Binary quadratic programming	Ding et al. [6, 7, 35]	Minimize the number of ungated flights and the total walking distances or connection times	Theoretical

Clique partitioning problem (CPP)	Dorndorf et al. [8]	(i) Maximizing the total assignment preference score (ii) Minimizing the number of unassigned flights (iii) Minimizing the number of tows (iv) Maximizing the robustness of the resulting schedule	Theoretical

Network representation	Maharjan and Matis [40]	Minimizing both fuel burn of aircraft and the comfort of connecting passengers	Real case (Continental Airlines at George W. Bush Intercontinental Airport in Houston (IAH))

Robust optimization	Diepen et al. [41]	Maximizing the robustness of a solution to the gate assignment problem	Real case (Amsterdam Airport Schiphol)

**Table 2:** Resolution methods.

Method	References	Approach/results	Problem type
Exact algorithms	Mangoubi and Mathaisel [11]	Linear programming relaxation	Real case (Toronto International Airport)
	Bihr [12]	Primal-dual simplex	Theoretical
	Yan and Huo [2]	Simplex; branch and bound	Real case (Chiang Kai-Shek Airport)
	Bolat [30, 34]; Xu and Bailey [14]; Li [39]	Branch and bound	Real case (King Khaled International Airport, KSA); theoretical

Heuristic algorithms	Thengvall et al. [43]	Bundle algorithm approach	Theoretical
	Yan and Tang [10]	Heuristic approach embedded in a framework designed	Real case (Taiwan International Airport)
	Ding et al. [6, 35]	Greedy algorithm	Theoretical
	Lim et al. [24]	The Insert Move Algorithm, the Interval Exchange Move Algorithm, and a Greedy Algorithm	Theoretical
	Diepen et al. [25]	Column generation	Real case (Amsterdam Airport Schiphol)
	Dorndorf et al. [8]	Heuristic based on the ejection chain algorithm	Theoretical
	Mangoubi and Mathaisel [11]	Heuristic approach	Real case (Toronto International Airport)
	Vanderstraeten and Bergeron [28]	ADAP	Theoretical
	Yan et al. [29]	Greedy heuristics	Real case (Chiang Kai-Shek Airport)
	Bolat [30]	Heuristic branch and trim	Real case (King Khaled International Airport, KSA)
	Bolat [34]	Heuristic branch and bound, SPH heuristic	Real case (King Khaled International Airport, KSA)
	Haghani and Chen [13]	Heuristic approach	Theoretical
	Genç [42]	Ground time maximization heuristic, and idle time minimization heuristic	Theoretical and real case (Ataturk Airport of Istanbul, Turkey)
	B.A.C.o.E.B. Team and A.I.C.o.E. Team [17]	A hybrid heuristics algorithm guided by simulated annealing and greedy heuristic	Theoretical
	Bouras et al. [45]	Heuristic approach	Theoretical

Metaheuristic algorithms	Ding et al. [6, 35]	Tabu search	Theoretical
	Ding et al. [7]	Simulated annealing, hybrid of simulated annealing and tabu search	Theoretical
	Lim et al. [24]	TS algorithm and a memetic algorithm	Theoretical
	Hu and Di Paolo [36]	New genetic algorithm with uniform crossover	Theoretical
	Drexl and Nikulin [3]	Pareto simulated annealing	Theoretical
	Xu and Bailey [14]	Tabu search	Theoretical
	Bolat [31]	Genetic algorithm	Real case (King Khaled International Airport, KSA)
	Şeker and Noyan [9]	Tabu search algorithms	Theoretical
	Zheng et al. [33]	A tabu search algorithm and metaheuristic method	Real case (Beijing International Airport, China)
	Wei and Liu [16]	A hybrid genetic algorithm	Theoretical
	Gu and Chung [44]	Genetic algorithms approach	Theoretical
	Cheng et al. [23]	Genetic algorithm (GA), tabu search (TS), simulated annealing (SA), and a hybrid approach based on SA and TS	Real case (Incheon International Airport, South Korea)
	Bouras et al. [45]	Genetic algorithm (GA), tabu search (TS), and simulated annealing (SA)	Theoretical

OPL	Li [5, 32]	Optimization programming language (CPLEX)	Real case (Continental Airlines, Houston Gorge Bush Intercontinental Airport)
	Tang et al. [27]	Using CPLEX 10.0 solver concert with C language	Real case (Taiwan International Airport)
	Prem Kumar and Bierlaire [18]	Optimization programming language (OPL)	Theoretical
	Maharjan and Matis [40]	AMPL/CPLEX 11.2	Real case (Continental Airlines at George W. Bush Intercontinental Airport in Houston (IAH))

**Table 3:** Number of publications per year.

Year	Number of publications	References
1974	1	Steuart [1]
1985	1	Mangoubi and Mathaisel [11]
1988	1	Vanderstraeten and Bergeron [28]
1990	1	Bihr [12]
1998	1	Haghani and Chen [13]
1999	2	Bolat [30], Gu and Chung [44]
2000	1	Bolat [34]
2001	3	Bolat [31], Xu and Bailey [14], Yan and Huo [2]
2002	2	Lam et al. [15], Yan et al. [29]
2003	1	Thengvall et al. [43]
2004	2	Ding et al. [6, 35]
2005	3	Ding et al. [7], Lim et al. [24], Al-Khalifah [19]
2007	4	Diepen et al. [25], Dorndorf et al. [20, 22], Yan and Tang [10]
2008	4	Diepen et al. [26], Drexl and Nikulin [3], Li [5, 32]
2009	4	Wei and Liu [16], Hu and Di Paolo [36], Tang et al. [27], B.A.C.o.E.B. Team and A.I.C.o.E. Team [17]
2010	4	Dorndorf et al. [8], Genç [42], Zheng et al. [33], Li [39]
2011	2	Prem Kumar and Bierlaire [18], Genç et al. [38]
2012	5	Li [39], Şeker and Noyan [9], Diepen et al. [41], Maharjan and Matis [40], Kaliszewski and Miroforidis [37], Cheng et al. [23]
2014	1	Bouras et al. [45]

**Table 4:** Number of publications per research area.

Area	Number of publications	References
Integer linear programming (IP)	3	Lim et al. [24], Diepen et al. [25], Diepen et al. [26]

Binary integer programming	6	Mangoubi and Mathaisel [11], Yan et al. [29], Vanderstraeten and Bergeron [28], Bihr [12], Tang et al. [27], Prem Kumar and Bierlaire [18]

Mixed integer linear programming (MILP)	3	Bolat [30], Bolat [31], Kaliszewski and Miroforidis [37]

Mixed integer nonlinear programming	3	Li [5, 32], Bolat [31]

Quadratic programming (QP)	6	Bolat [34], Zheng et al. [33], Xu and Bailey [14], Ding et al. [6, 7, 35]

Multiple objective GAP formulations	5	Hu and Di Paolo [36], Wei and Liu [16], B.A.C.o.E.B. Team and A.I.C.o.E. Team [17], Yan and Huo [2], Kaliszewski and Miroforidis [37]

Stochastic models	3	Yan and Tang [10], Genç et al. [38], Şeker and Noyan [9]

Quadratic assignment problem (QAP)	2	Drexl and Nikulin [3], Haghani and Chen [13]

Scheduling problems	1	Li [39]

Clique partitioning problem (CPP)	1	Dorndorf et al. [8]

Network representation	1	Diepen et al. [41]

Robust optimization	1	Maharjan and Matis [40]

Exact algorithms	7	Mangoubi and Mathaisel [11], Bihr [12], Yan and Huo [2], Bolat [30, 34]; Xu and Bailey [14]; Li [39]

Heuristic algorithms	16	Thengvall et al. [43], Yan and Tang [10], Ding et al. [6, 35], Lim et al. [24], Diepen et al. [25], Dorndorf et al. [8], Mangoubi and Mathaisel [11], Vanderstraeten and Bergeron [28], Yan et al. [29], Bolat [30, 34], Haghani and Chen [13], Genç [42], B.A.C.o.E.B. Team and A.I.C.o.E. Team [17], Bouras et al. [45]

Metaheuristic algorithms	14	Ding et al. [6, 35], Ding et al. [7], Lim et al. [24], Hu and Di Paolo [36], Drexl and Nikulin [3], Xu and Bailey [14], Bolat [31], Şeker and Noyan [9], Zheng et al. [33], Wei and Liu [16], Gu and Chung [44], Cheng et al. [23], Bouras et al. [45]

OPL	4	Li [5, 32], Tang et al. [27], Prem Kumar and Bierlaire [18], Maharjan and Matis [40]
